# Mortality-associated plasma proteome dynamics in a prospective multicentre sepsis cohort

**DOI:** 10.1016/j.ebiom.2024.105508

**Published:** 2024-12-15

**Authors:** Lars Palmowski, Maike Weber, Malte Bayer, Yuxin Mi, Karin Schork, Martin Eisenacher, Hartmuth Nowak, Tim Rahmel, Lars Bergmann, Andrea Witowski, Björn Koos, Katharina Rump, Dominik Ziehe, Ulrich Limper, Dietrich Henzler, Stefan Felix Ehrentraut, Alexander Zarbock, Roman Fischer, Julian C. Knight, Michael Adamzik, Barbara Sitek, Thilo Bracht

**Affiliations:** a Klinik für Anästhesiologie, Intensivmedizin und Schmerztherapie, Universitätsklinikum Knappschaftskrankenhaus Bochum, Bochum, Germany; b Medizinisches Proteom-Center, Medical Faculty, Ruhr University Bochum, Bochum, Germany; c Center for Protein Diagnostics (PRODI), Medical Proteome Analysis, Ruhr University Bochum, Bochum, Germany; d CUBiMed.RUB, Core Unit Bioinformatics, Medical Faculty, Ruhr University Bochum, Bochum, Germany; e Center for Artificial Intelligence, Medical Informatics and Data Science, University Hospital Knappschaftskrankenhaus Bochum, Bochum, Germany; f Daxing Research Institute, University of Science and Technology Beijing, Beijing, China; g Department of Anesthesiology and Operative Intensive Care Medicine, University of Witten/Herdecke, Cologne Merheim Medical School, Cologne 51109, Germany; h Department of Anesthesiology, Surgical Intensive Care, Emergency and Pain Medicine, Ruhr-University Bochum, Klinikum Herford, Herford 32049, Germany; i Klinik für Anästhesiologie und Operative Intensivmedizin, Universitätsklinikum Bonn, Bonn 53127, Germany; j Department of Anesthesiology, Intensive Care and Pain Medicine, University Hospital of Münster, Münster, Germany; k Target Discovery Institute, Nuffield Department of Medicine, University of Oxford, Oxford OX3 7FZ, UK; l Chinese Academy of Medical Science Oxford Institute, University of Oxford, Oxford OX3 7BN, UK; m Wellcome Centre for Human Genetics, Nuffield Department of Medicine, University of Oxford, Oxford OX3 7BN, UK; n NIHR Oxford Biomedical Research Centre, Oxford OX3 9DU, UK

**Keywords:** Proteomics, Sepsis, Machine learning, Feature importance, Mass spectrometry

## Abstract

**Background:**

Sepsis remains a leading cause of mortality in intensive care units. Understanding the dynamics of the plasma proteome of patients with sepsis is critical for improving prognostic and therapeutic strategies.

**Methods:**

This prospective, multicentre observational cohort study included 363 patients with sepsis recruited from five university hospitals in Germany between March 2018 and April 2023. Plasma samples were collected on days 1 and 4 after sepsis diagnosis, and proteome analysis was performed using mass spectrometry. Classical statistical methods and machine learning (random forest) were employed to identify proteins associated with 30-day survival outcomes.

**Findings:**

Out of 363 patients, 224 (62%) survived, and 139 (38%) did not survive the 30-day period. Proteomic analysis revealed significant differences in 87 proteins on day 1 and 95 proteins on day 4 between survivors and non-survivors. Additionally, 63 proteins were differentially regulated between day 1 and day 4 in the two groups. The identified protein networks were primarily related to blood coagulation, immune response, and complement activation. The random forest classifier achieved an area under the receiver operating characteristic curve of 0.75 for predicting 30-day survival. The results were compared and partially validated with an external sepsis cohort.

**Interpretation:**

This study describes temporal changes in the plasma proteome associated with mortality in sepsis. These findings offer new insights into sepsis pathophysiology, emphasizing the innate immune system as an underexplored network, and may inform the development of targeted therapeutic strategies.

**Funding:**

10.13039/501100008530European Regional Development Fund of the 10.13039/501100000780European Union. The State of North Rhine-Westphalia, Germany.

**Research in context**

**Evidence before this study**

Personalised medicine requires an accurate knowledge of the molecular processes that underly a disease and its clinical manifestations, but little is known about the plasma proteome dynamics in patients with sepsis and their association with survival. Previous research has identified plasma proteins that were predictive of outcome, but these studies mostly relied on single-time-point measurements, missing the temporal changes occurring over the course of the disease. Until now, machine learning has been used to predict outcome in sepsis based primarily on clinical and laboratory parameters, while it was not applied to proteomics data and used to identify relevant proteins. The analysis of time-resolved proteomics data using univariate statistics and machine learning is still fragmentary, leaving an opportunity to better understand the molecular landscape of sepsis.

**Added value of this study**

In this study, temporal plasma proteome dynamics in critically ill patients with sepsis were investigated using high-throughput mass-spectrometry in combination with univariate statistics and machine learning. We provide a comprehensive view of proteome changes associated with 30-day survival and disease severity and identified key pathophysiological processes, such as tissue damage, coagulation disorders, and the crucial yet underappreciated role of complement activation. By tracking the plasma proteome in early sepsis, we found that temporal changes were generally associated with worse outcome and identified the innate immune system as a key player that is underrepresented in clinical practice.

**Implications of all the available evidence**

Our study highlights the relevance of the innate immune system and especially complement activation in sepsis severity and mortality. The complement system, however, is not adequately represented in standard diagnostics and should be integrated into clinical routine. We highlight the well-known process of blood coagulation, which calls for further research to allow the development of appropriate therapies. Our research paves the way for future studies to explore targeted interventions that address the underlying innate immune and coagulation dysregulations in sepsis, potentially improving patient outcomes.

## Introduction

Sepsis, a life-threatening organ dysfunction caused by a dysregulated host response to an infection, remains a significant challenge in critical care medicine.^1^ Despite advancements in therapeutic strategies and supportive care, the prognosis for patients with sepsis remains poor and difficult to predict.^2^ Traditional clinical markers and scoring systems, such as the Sequential Organ Failure Assessment (SOFA) score, offer limited insight into the complex biological processes driving sepsis and its outcomes.^3^^,^^4^

In the era of precision medicine, having an accurate and comprehensive understanding of the pathophysiology of diseases is crucial for the development of personalised therapies, particularly for syndromes as complex as sepsis.^5^ In recent years, the analysis of the plasma proteome has emerged as a promising approach to unravel the complex biological processes underlying sepsis and to identify potential therapeutic targets and biomarkers for prognosis.^6^, ^7^, ^8^, ^9^ While a single measurement of the plasma proteome already provides insights, monitoring the trajectory of proteomic changes over the course of the illness may offer additional understanding of the biological processes involved.^10^, ^11^, ^12^ This is supported by the advancements in mass spectrometry and data processing that have significantly improved the analysis of the plasma proteome providing an increasing amount of information.^13^ In addition, recent developments in machine learning have shown great potential in identifying nonlinear relationships within big data, facilitating the analysis of these increasingly complex datasets, such as those generate by high-throughput proteomics.^14^, ^15^, ^16^, ^17^

However, a comprehensive characterisation of plasma proteome dynamics in critically ill patients with sepsis and their association with survival has yet to be achieved. Therefore, we applied univariate statistical methods and machine learning techniques to identify and characterise the proteomic changes at two time points within the early course of the disease as analysed by liquid chromatography-tandem mass spectrometry (LC-MS/MS) in a large, prospective, multi-centre cohort. Finally, we tested the hypothesis that specific alterations and protein networks in the plasma proteome of patients with sepsis are associated with survival outcomes.

## Methods

### Study design and conceptual overview

As part of the multicentric, prospective, observational SepsisDataNet.NRW and CovidDataNet.NRW studies, 363 patients with newly diagnosed sepsis (<48 h) according to the Sepsis-3 definition^1^ were enroled at five university hospitals in Germany from March 2018 to April 2023 (German Clinical Trial Registry Nos. DRKS00018871 and DRKS00026184). For all patients included, written informed consent was obtained. The patient cohort consisted of both surgical and medical cases admitted to the Intensive Care Unit (ICU). The exclusion criteria were as follows: (1) individuals younger than 18 years at the time of ICU admission, (2) refusal or retraction of consent, and (3) cessation of treatment. The subgroup of critically ill COVID-19 patients was further identified through a positive PCR test after admission. Plasma samples were collected at the day of study inclusion (day 1) and at day 4 to monitor relevant changes from the onset of sepsis to early disease progression. Only patients with a clear 30-day survival status were included in the analysis. Sex was considered as reported by health ensurances. The proportion of missing values in patient data is available in Table 1. The treatment of the patients was performed under the responsibility of the attending physicians and based on prevailing national and international guidelines as well as in-house standards and was not influenced by study participation.^18^^,^^19^ All research was conducted in accordance with the revised Declaration of Helsinki. The primary outcome was the 30-day survival. Parts of the cohort have been previously analysed and published in relation to other research questions.^20^^,^^21^

**Table 1:** Baseline characteristics of the multicentric sepsis cohort (n = 363).

Variable	Overall, n = 363	Missing values, n (%)	Survivor, n = 224^a^	Missing values, n (%)	Non-Survivor, n = 139^a^	Missing values, n (%)	p-value
Age, yrs. (IQR)	64 (55.0–74.5)	29 (8.0%)	63 (58.5–77.5)	21 (9.4%)	67 (53.0–72.0)	8 (5.8%)	**0.002**
Male sex, n (%)	130 (36%)	29 (8.0%)	80 (36%)	21 (9.4%)	50 (36%)	8 (5.8%)	0.909
Inclusion SOFA Score (IQR)	9 (6–12)	0 (0%)	8 (6–11)	0 (0%)	9 (6–12)	0 (0%)	0.489
Inclusion SAPSII Score (IQR)	41 (31–52)	43 (11.8%)	40 (28–51)	37 (16.5%)	43.0 (33.0–55.0)	6 (4.3%)	0.601
ICU length of stay, days (IQR)	8 (3.0–17.0)	42 (11.6%)	9.0 (3.0–24.0)	37 (16.5%)	8.0 (3.0–15.0)	6 (4.3%)	0.096
Critical Covid-19 Infection^a^, n (%)	83 (23%)		48 (21%)		35 (25%)		0.441
Comorbid condition, n (%)							
Hypertension	236 (65%)	6 (1.7%)	148 (66%)	4 (1.8%)	88 (63%)	2 (1.4%)	0.567
Chronic kidney disease	77 (21%)	6 (1.7%)	56 (25%)	4 (1.8%)	21 (15%)	2 (1.4%)	**<0.001**
COPD^b^	42 (12%)	6 (1.7%)	31 (13%)	4 (1.8%)	11 (8%)	2 (1.4%)	0.533
Diabetes mellitus	104 (29%)	6 (1.7%)	67 (30%)	4 (1.8%)	37 (27%)	2 (1.4%)	0.550
Obesity	108 (30%)	6 (1.7%)	65 (29%)	4 (1.8%)	43 (31%)	2 (1.4%)	0.724
Cardiovascular disease	131 (36%)	6 (1.7%)	80 (36%)	4 (1.8%)	51 (37%)	2 (1.4%)	0.910
Malignancy	77 (21%)	6 (1.7%)	41 (18%)	4 (1.8%)	37 (27%)	2 (1.4%)	0.066
Organ transplantation	42 (12%)	6 (1.7%)	32 (14%)	4 (1.8%)	1 (7%)	2 (1.4%)	**0.043**
Lab values, day 1, (IQR)							
C-reactive protein, mg/dL	13.17 (7.57–25.24)	15 (42.6%)	13.17 (8.01–25.24)	87 (38.8%)	13.23 (6.80–23.92)	68 (48.9%)	0.800
Procalcitonin, ng/mL	0.76 (0.20–6.20)	160 (44.1%)	0.50 (0.19–4.50)	92 (41.1%)	1.18 (0.24–10.29)	68 (48.9%)	0.200
Lactate, mmol/L	2.0 (1.5–3.2)	153 (42.1%)	1.7 (1.3–2.4)	8 (38.4%)	2.7 (1.9–5.9)	67 (48.2%)	**<0.001**
White blood cells, n/μL	11.20 (7.78–16.73)	156 (43.0%)	9.45 (7.28–14.60)	88 (39.3%)	15.55 (9.03–19.06)	68 (48.9%)	**<0.001**
Platelet count, n/mL	201.0 (128–283)	148 (40.8%)	212.5 (137–294)	88 (39.3%)	176.2 (118–262)	60 (43.2%)	0.080
Quick value, %	72.0 (63.0–83.0)	149 (41.0%)	76.0 (67.5–89.0)	88 (39.3%)	71.0 (57.0–79.0)	61 (43.9%)	**0.003**
PTT^c^, s	42.8 (37.6–54.7)	151 (41.6%)	41.4 (36.8–49.5)	8 (38.4%)	45.1 (38.5–61.8)	65 (46.8%)	**0.011**

Bold indicates statistically significant values (p < 0.05).

a As described in methods section.

b Chronic obstructive lung disease.

c Partial thromboplastin time.

### Plasma proteomics

A detailed description of the applied proteomics methods can be found as Supplementary material. Briefly, 1 μL of plasma per sample was digested using the SP3 protocol as described previously.^20^ 579 samples from 363 patients were analysed distributed over eight batches using three different LC-MS setups. Batch S3 was measured using an Ultimate 3000 RSLCnano HPLC coupled to an Orbitrap Fusion Lumos mass spectrometer. Batches C3 and S9 were analysed on a Vanquish Neo UHPLC coupled to an Orbitrap Exploris 240. All other batches were analysed on an Ultimate 3000 RSLCnano HPLC coupled to an Orbitrap Exploris 240 (all Thermo Scientific, Bremen, Germany). The mass spectrometers were operated in data-independent acquisition mode and raw data were processed using DIA-NN (v.1.8.1) searching the UniProt/SwissProt database restricted to *Homo sapiens* (v.2022_05). All batches were processed separately. Subsequently, batch effects were normalised and evaluated as described previously^17^ (Supplementary Methods and Figs. S1 and S2). Functional annotation and enrichment analyses were performed using the STRING web interface (string-db.org, v.12.0)^22^ and the interaction-network was edited with Cytoscape (v.3.10.2).

### Machine learning classifiers

Complementary to univariate analysis, different machine learning models were developed to predict the primary outcome (30-day survival) as well as the 4-day survival, respectively. To this end, three prediction scenarios were developed:

Prediction of 30-day survival based on proteome data from day 1 (n = 334, n_s_ = 204, n_d_ = 130)

Prediction of 4-day survival based on proteome data from day 1 (n = 334, n_s_ = 283, n_d_ = 51)

Prediction of 30-day survival using proteome data from day 1 and day 4 as well as day 4/ day 1 ratios (n = 216, n_s_ = 132, n_d_ = 84)

Where n_s_ is the number of surviving patients and n_d_ is the number of deceased patients. Support vector machine (SVM), Logistic regression combined with elastic net or lasso regularization, gradient boosted regression trees and random forest models were applied on each prediction scenario. Additionally for the random forest models hyperparameters were tuned and can be found in Supplementary Table S1. Proteins with more than 30% missing values were removed, resulting in 266 remaining proteins for the prediction based on data from day 1 and 266 proteins plus their ratios based on the information of both day 1 and day 4. Each dataset was randomly split into training and test sets for 100 times to perform Monte Carlo cross-validation (MCCV)^23^ and robustly estimate the model performance. Missing values were imputed with the median of a feature after each data-split. Other approaches, such as random forest imputation, were too time-intensive to calculate in combination with MCCV. For the prediction of 4-day survival, a resampling with replacement was performed to reduce the number of samples in the larger class (survivors) down to n_s_ = 100, to avoid a classification bias. Analyses were performed in python (v.3.11.5) using pandas (v.2.1.1) and scikit-learn (v.1.4.1). SHapley Additive exPlanations (SHAP; shap package v.0.42.1)^24^ were used to evaluate the feature’s contribution to the machine learning models. Each feature was given a rank based on its SHAP value for each iteration of the MCCV and the median rank was used to represent the feature importance.

### Statistics

Continuous variables are presented as means ± standard deviation (SD) for normally distributed data and as medians with interquartile ranges (IQR, 25th; 75th percentile) for non-normally distributed data. Group differences of baseline characteristics were examined using Student’s t-test or Wilcoxon rank-sum test for continuous variables and the Chi-square test or Fisher's exact test for categorical variables. For non-parametric analyses, the Kruskal–Wallis test followed by Dunn’s test with Bonferroni correction was used. A p-value of less than 0.05 was considered statistically significant. Confidence intervals (CI) were calculated with 95% coverage. Differences in protein intensities between patient groups were tested for significance using Student’s t-test. Proteins with at least five observations per condition were considered for testing and p-values were corrected using the Benjamini-Hochberg method. Ratios of mean intensities were calculated based on delogarithmised intensities. Proteins with a pFDR value ≤ 0.05 were considered significant. Differences in variance were assessed using Levene’s test. Linear regression analysis was calculated using linear models from R stats. Statistical analyses were performed using R (v.4.4). The external cohort of patients with sepsis was analysed using a linear model considering age and sex as covariables as described before.^9^

### Role of funders

The funders had no role in the design, analysis, interpretation or publication of the study.

### Ethics approval

The study was reviewed and approved by the Ethics Committee of the Medical Faculty at Ruhr-University Bochum, Germany (approval no. 5047-14 and 18-6606-BR).

## Results

### Cohort description

The study included 363 patients with sepsis, of which 224 (62%) survived and 139 (38%) did not survive the 30-day period (Table 1). The median age was 64 years (IQR 55.0–74.5), with survivors having a median age of 63 years (IQR 58.5–77.5) and non-survivors 67 years (IQR 53.0–72.0, p = 0.002). The median SOFA score at admission was 9 (IQR 6–12), with similar scores in survivors (8, IQR 6–11) and non-survivors (9, IQR 6–12, p = 0.489). Critical COVID-19 infection was present in 23% of the cohort, with no significant difference between survivors (21%) and non-survivors (25%, p = 0.441).

Hypertension was the most common comorbidity, present in 65% of patients, with no significant difference between survivors (66%) and non-survivors (63%, p = 0.567). Chronic kidney disease was more prevalent in survivors (25%) than in non-survivors (15%), p < 0.001). Other comorbidities such as COPD, diabetes mellitus, obesity, and cardiovascular disease showed no significant differences between groups (each p > 0.05). Laboratory values on day 1 showed that lactate concentrations were significantly higher in non-survivors (2.7 mmol/L, IQR 1.9–5.9) compared to survivors (1.7 mmol/L, IQR 1.3–2.4, p < 0.001). White blood cell counts were also significantly higher in non-survivors (15.55/μL, IQR 9.03–19.06) compared to survivors (9.45/μL, IQR 7.28–14.60, p < 0.001). C-reactive protein (CRP) and procalcitonin concentrations did not differ significantly between the groups.

### Plasma proteome dynamics

**Fig. 1:**
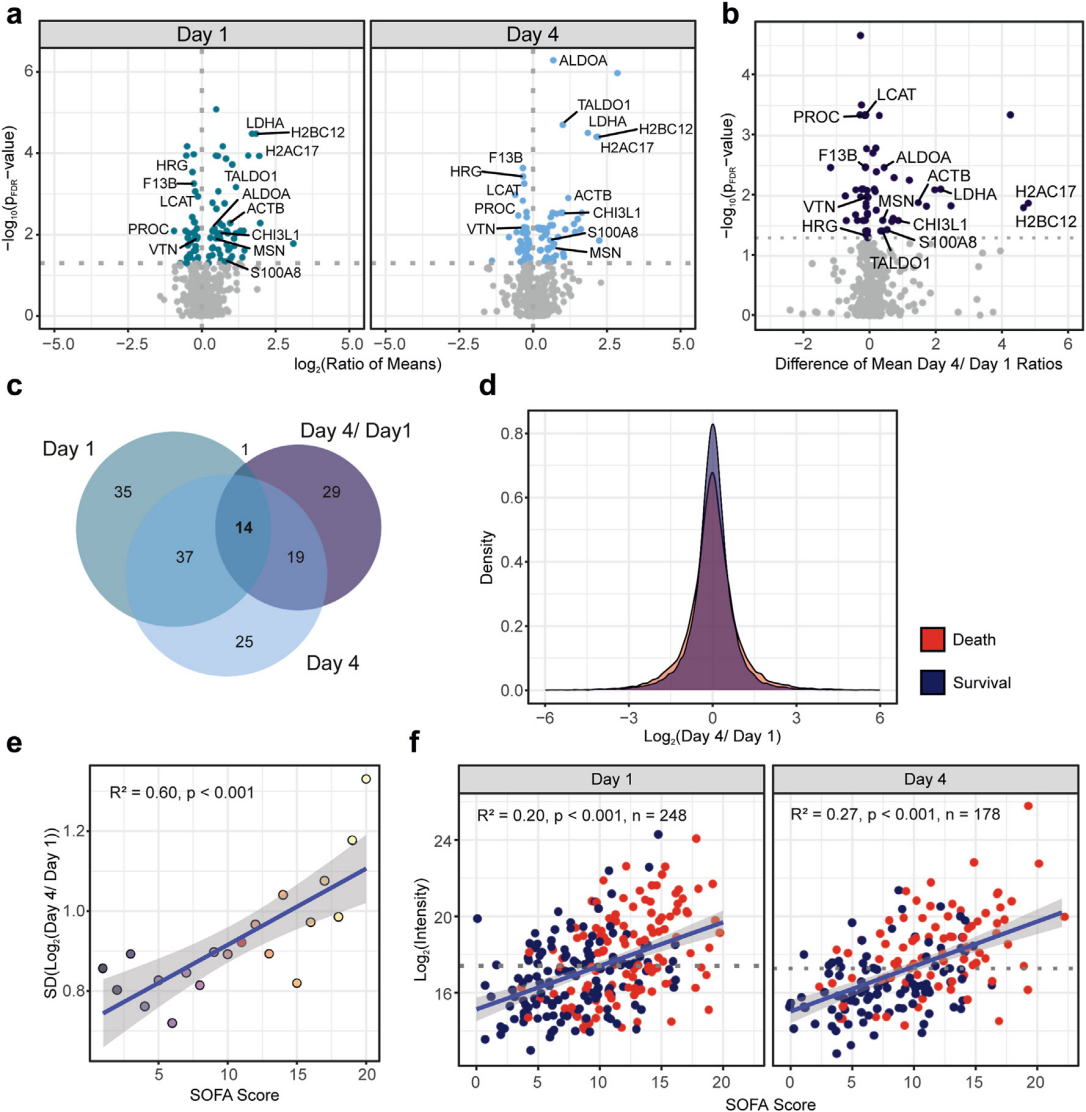
Proteome analysis according to 30-day survival. **(a)** Volcano plots representing the statistical analysis of survived and deceased patients for day 1 and day 4, respectively (Death/Survival). Coloured proteins passed the significance threshold of p_FDR_ value ≤ 0.05 (t-test, Benjamini-Hochberg corrected). **(b)** Volcano plot illustrating the statistical analysis of survived and deceased patients based on day 4/day 1 ratios. Differences calculated as mean ratio death – mean ratio survival. Coloured proteins passed the significance threshold of p_FDR_ value ≤ 0.05 (t-test, Benjamini-Hochberg corrected). **(c)** Venn diagram illustrating significant proteins for day 1, day 4 and the day 4/day 1 ratios. The 14 proteins in the intersect of all comparisons are labelled with gene names in figure parts a and b. **(d)** Density plots representing distribution of day 4/day 1 ratios for survived and deceased patients. The variance within both patient groups was found significantly different (Levene test, p < 0.0001). **(e)** Linear regression of the standard deviation of day 4/day 1 ratios with the SOFA score. Blue line representing the linear fit with its confidence interval. **(f)** Linear regression analysis of Myoglobin (MB) with the SOFA score separately for days 1 and 4. Blue and red data points represent survived and deceased patients, respectively.

We quantified 613 plasma proteins of which 87 and 95 were found to be significantly differentially abundant between deceased and survived patients at day 1 (n = 363 patients) and day 4 (n = 312 patients who survived at least 4 days), respectively (Fig. 1a, Supplementary Table S2). Furthermore, we calculated the relative changes of protein intensities between day 1 and day 4 and tested for differences in protein regulations between both survival groups (n = 216 patients with measurements for both time points). Here, 63 proteins showed a significant difference in their dynamics (Fig. 1b, Supplementary Fig. S3). The overlap of all three comparisons was 14 proteins that were associated with tissue damage (LDHA, H2BC12, H2BC17, ACTB; for ease of reading, the full names of the proteins are given in Table 2, Fig. 1c, Supplementary Table S3), but also with blood coagulation (HRG, F13B, PROC) and immune response (CHI3L1, S100A8). In addition, glycolytic enzymes were apparent (ALDOA, TALDO1), that hinted towards a metabolic shift towards anaerobic glycolysis. As a global observation, we found a striking relation of temporal protein dynamics with survival and overall greater changes – irrespective of their direction - in deceased patients and the variance in both subgroups differed significantly (Fig. 1d, p < 0.001). Using the standard deviation as a measure, we found a significant correlation of day 1 – day 4 proteome dynamics with disease severity as represented by the SOFA score (Fig. 1e). On the level of individual proteins, we found, despite considerable inter-individual variation, many examples of significant linear correlations of protein intensities with the SOFA scores, as illustrated by MB (Fig. 1f, further examples presented in Supplementary Fig. S4).

**Table 2:** Gene and protein names mentioned in the text.

Gene name	Protein name
ACTB	Actin, cytoplasmic 1
ALDOA	Fructose-bisphosphate aldolase A
ALDOB	Fructose-bisphosphate aldolase B
CHI3L1	Chitinase-3-like protein 1
F13B	Coagulation factor XIII B chain
F5	Coagulation factor V
FETUB	Fetuin-B
H2AC17	Histone H2A type 1
H2BC12	Histone H2B type 1-K
HRG	Histidine-rich glycoprotein
KNG1	Kininogen-1
LCAT	Phosphatidylcholine-sterol acyltransferase
LCP1	Plastin-2
LDHA	L-lactate dehydrogenase A chain
MB	Myoglobin
PROC	Vitamin K-dependent protein C
S100A8	Protein S100-A8
S100A9	Protein S100-A9
TALDO1	Transaldolase
TKT	Transketolase
VWF	von Willebrand factor

### Machine learning

We tested five machine learning models for survival prediction: Support vector machine (SVM), Logistic regression combined with elastic net or lasso regularization, gradient boosted regression trees and random forest. While the models showed an overall similar performance, the random forest classifier consistently delivered the highest area under the receiver operating characteristic (AUROC) and Matthew’s correlation coefficient evaluation metrics, although the logistic regression model with elastic showed a promising performance as well (Table 3, Supplementary Table S4). The highest AUROC was 0.77 and achieved by the classifier for prediction of 4-day survival. As expected, the prediction of 30-day survival at day 1 was more challenging and the respective classifier achieved an AUROC of 0.70. The prediction of 30-day survival could be improved when all data (day 1, day 4, day 4/day 1 ratios) were considered and the respective classifier showed an AUROC of 0.75. Due to its performance and know robustness towards overfitting, we used the random forest classifier for feature importance analysis.

**Table 3:** Mean performance of the Monte Carlo cross-validation of the three random forest classifiers.

Metric (mean)	30-day survival based on data from day 1 (n_s_ = 204, n_d_ = 130)	4-day survival based on data from day 1 (n_s_ = 283, n_d_ = 51)	30-day survival based on data from days 1 and 4 (n_s_ = 132, n_d_ = 84)
AUROC	0.70 (±1.28%)	0.77 (±1.68%)	0.75 (±1.33%)
Sensitivity	0.57 (±2.74%)	0.53 (±4.81%)	0.59 (±3.46%)
Specifity	0.70 (±1.9%)	0.84 (±1.54%)	0.76 (±2.04%)
Precision	0.55 (±1.9%)	0.65 (±3.18%)	0.61 (±2.17%)
Accuracy	0.65 (±1.27%)	0.73 (±1.42%)	0.69 (±1.3%)

**Fig. 2:**
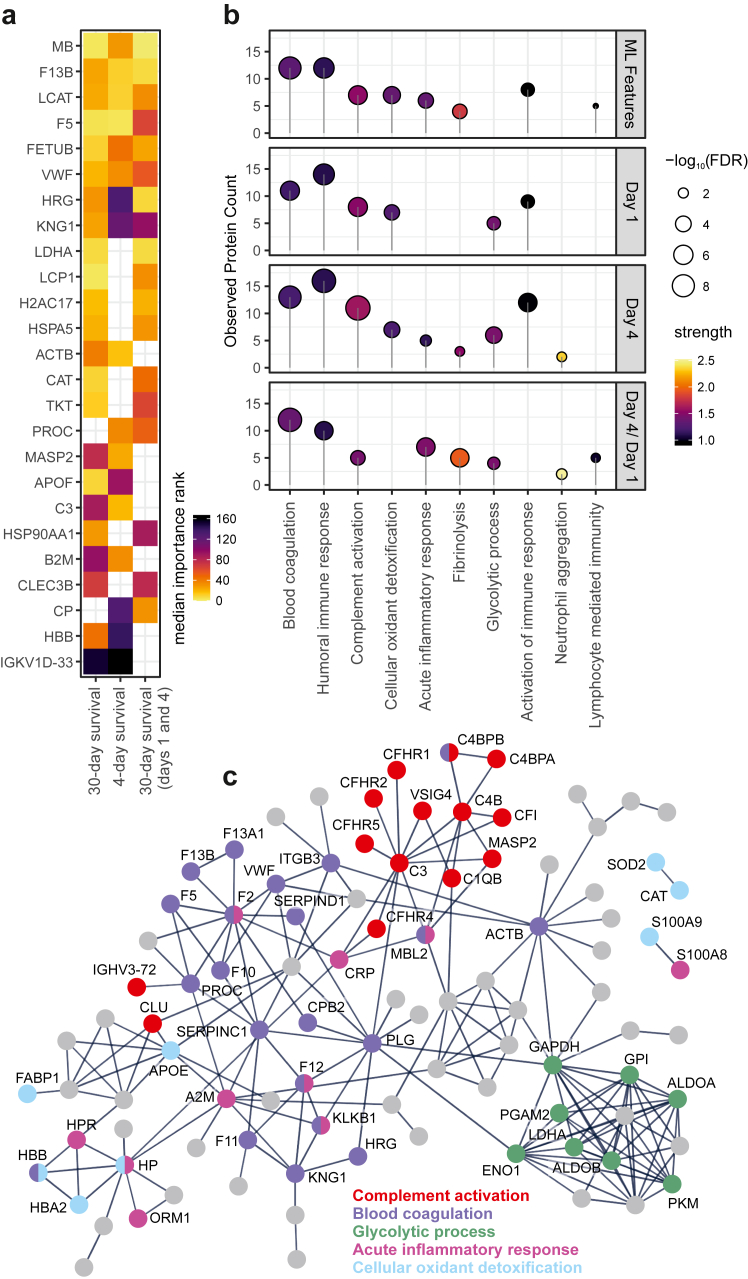
Machine Learning and Functional Protein Analysis. **(a)** Heatmap illustrating the 25 most frequently selected ML features. Three independent Random Forest classifiers were trained 100 times each and the selected features were interpreted using Shapley Additive Explanations (SHAP). Colour representing the median importance rank **(b)** Functional enrichment analysis using Gene Ontology (GO) Biological Processes. Ten selected categories illustrated for analysis using all selected ML features as well as the significant proteins for day 1, day 4 and day 4/day 1 ratios. **(c)** Protein interaction network illustrating major groups of proteins associated with 30-day survival. All differentially abundant proteins were analysed using STRING and significantly enriched biological processes were highlighted. Only interactions with highest confidence displayed, disconnected nodes not shown, highlighted proteins labelled with gene names.

We applied Shapley feature importance to identify the proteins that had a large impact on the predictions of the classifiers (Supplementary Table S5). Proteins with repeatedly high importance and low ranks in the Monte Carlo cross-validation carried relevant information for survival prediction and might therefore also indicate associated disease mechanisms (Fig. 2a). This could exactly be observed for the top eight proteins showing high importance in all trained classifiers, five of which were related to blood coagulation (F13B, F5, VWF, HRG, KNG1). Other important disease-relevant processes such as liver failure (LCAT, LDHA), complement activation (C3) and tissue damage (MB, H2AC17, ACTB) were also represented by the ML features. Overall, the important features were complementary to univariate statistics, with 35 proteins (43%) that were not found to be significant in any univariate test. Interestingly, for 30-day survival classification based on day1 and day 4, for six proteins (F5, FETUB, LCP1, LDHA, MB, TKT) intensities of both time points were found with high importance, thus underlining their robust association with survival in sepsis, irrespective of the investigated time points.

### Functional analysis

**Fig. 3:**
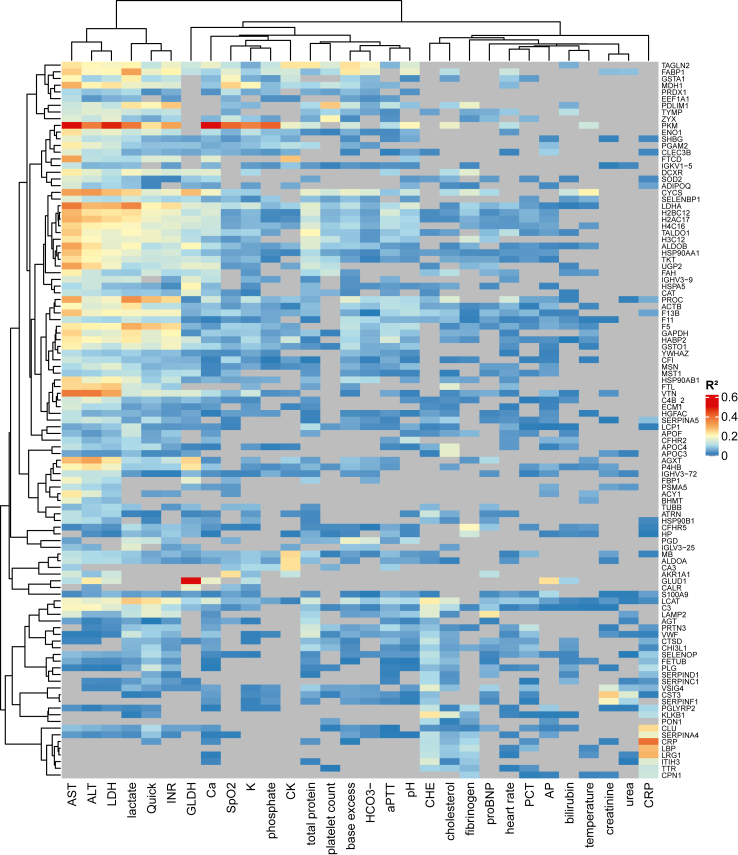
Correlation of protein intensities with clinical parameters. Heatmap illustrating the linear regression analysis of baseline characteristics with protein intensities at day 1. All proteins found to be significantly differentially abundant in a univariate analysis were considered for analysis. Colours represent the R^2^ of the linear models for each pair. Models that did not pass a significance threshold of p < 0.05 displayed in grey; hierarchical clustering using Pearson correlation and complete linkage.

We performed annotation and enrichment analysis based on gene ontologies separately for all proteins with high importance in ML (81 proteins), and univariate testing for days 1 and 4, as well as day 1 to day 4 proteome dynamics. All sets of proteins were significantly enriched in proteins associated with blood coagulation, humoral immune response and complement activation (Fig. 2b). These categories represented the two major classes of proteins that were found to be differentially abundant between deceased and survived patients, namely complement and coagulation factors (Fig. 2c). Another major class of differential proteins were associated with liver failure and mostly consisted of enzymes related to glucose metabolism. To substantiate these observations, we performed linear regression of all significantly differential proteins with the available clinical variables. Most significant correlations were found with liver values AST, ALT and LDH, thus underlining the relevance of liver damage for our observations (Fig. 3). Lactate, QUICK and INR also showed significant associations with several proteins that had a remarkable overlap with those correlating with liver values, therefore pointing towards an impaired liver synthesis. Smaller sets of proteins could be allocated to specific biological processes, such as the alarmins S100A8/A9 which are released by activated neutrophils and were found at day 4 as well as to be significantly upregulated in deceased patients between day 1 and 4.

### Comparison with an external sepsis cohort

**Fig. 4:**
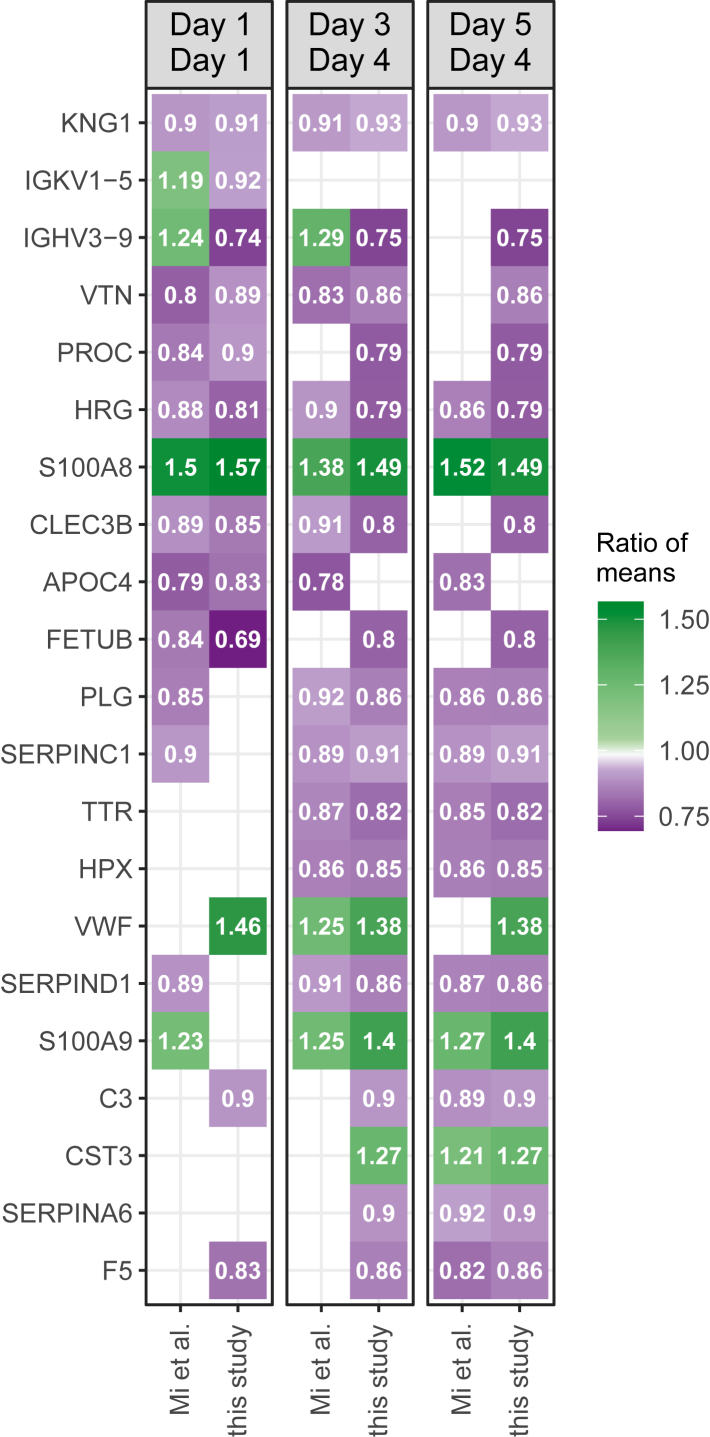
Comparison and validation using an independent sepsis cohort. Heatmap illustrating the comparison of this study with the study of Mi et al.^9^ Colour coded ratios of mean intensities (RoM, calculated Death/Survival) are shown for both studies. Analysis according to 30-day survival was done for days 1 and 4 (this study) and days 1, 3 and 5 (Mi et al.). Column titles indicate the respective comparisons. All displayed genes passed an p_FDR_ value ≤ 0.05 threshold in both studies in at least one of the three comparisons; all displayed RoM had a significant corresponding p_FDR_-value.

To evaluate to which extent our results can be generalised and applied to other cohorts, we compared them with a large, comprehensive data set that was recently described by Mi et al.^9^ To allow comparability, the authors of the study re-analysed their cohort of n = 1173 patients with sepsis according to 30-day survival (n = 972 survivors and n = 201 non-survivors). The 30-day mortality in the cohort was 17.1% with a median SOFA score of 6 (IQR 3–8). The analysis between survivors and non-survivors was calculated for days 1, 3 and 5 of ICU stay (Supplementary Table S6) and the numbers of significantly differentially abundant proteins were 43, 31 and 35, respectively (p_FDR_ value ≤ 0.05, Supplementary Table S7). Of these proteins, 37, 26 and 33 were also quantified in our study. We compared the results from day 1 between the two studies as well as our results from day 4 against their corresponding results from day 3 or day 5. We found 10 (27%), 13 (50%) and 13 (39%) proteins to be significantly differentially abundant in both studies for the respective comparisons. Notably, except for two immunoglobulins, the relative differences in protein abundances between survived and deceased patient were in agreement between the studies (Fig. 4).

## Discussion

With the present study, we aimed to comprehensively characterise the plasma proteome dynamics related to survival and mortality in sepsis. Significant differences were evident already on the day of sepsis diagnosis (day 1, 87 proteins), although being slightly more pronounced at day 4 (95 proteins). Together, 111 proteins were found to be differentially abundant at both time points, representing roughly a fifth of the measured proteins. Furthermore, we observed major differences between the survival groups for the proteome dynamics between both time points. Here, the peak width of aggregated protein regulations (day 4/day 1 ratios) correlated significantly with disease severity. Separate assessment of death and survival groups revealed a significantly wider distribution in deceased patients, reflecting overall more pronounced changes over time. Similar observations were previously made in a cohort of patients with sepsis receiving either ketogenic nutrition or standard diet.^25^ These observations highlight the sensitivity of the plasma proteome to the overall health status of patients with sepsis. Patients who exhibited substantial alterations in their plasma proteome tended to have worse outcomes, suggesting that these proteome changes reflect more severe or advanced disease states. These hypotheses could be substantiated for several proteins which showed a significant linear correlation with disease severity (Supplementary Fig. S1). Overall, our findings suggest that no “protective” changes in the plasma proteome occurred, but temporal changes were generally related to a worse outcome.

Within these overarching observations, we identified protein networks with differential expression that were significantly associated with 30-day survival outcomes (Fig. 2c). Notably, two major groups of proteins exhibited reduced abundance in deceased patients. The first group involves the coagulation system, which is well-characterised in sepsis, routinely assessed in clinical practice, and represented by surrogates in scores, such as the SOFA score.^26^ In contrast, the second group involves the complement system, a crucial component of the innate immune response. Unlike the coagulation system, the complement system has not yet been extensively explored for its prognostic or therapeutic potential in sepsis, highlighting a possible area for future research. Several corresponding proteins showed inverse correlation with the SOFA score, illustrating their consumption by activated complement and coagulation cascades. Contrary to previous observations,^27^ our data even suggests a linear inverse correlation of C3 with the SOFA score and found significantly lower levels in deceased patients. Proteins that showed higher abundance in deceased patients, on the other hand, were associated with the well-known processes of organ failure in sepsis, most prominently with liver failure (e.g., ALDOB, TALDO1, LDHA).^28^ The corresponding enzymes reflected the metabolic switch towards anaerobic glycolysis. Other proteins hinted towards cell death, but allowed only limited conclusions on the affected cell types due to their less specific tissue expression (e.g., MB, striated muscle cells; LCP1, immune cells).

To enhance the value of our findings with a complementary analytical technique, we incorporated a machine learning approach to predict mortality. While this method is already well-established for outcome prediction in sepsis,^29^, ^30^, ^31^ our analysis was based solely on the plasma proteome. Similar to studies that primarily relied on clinical or laboratory parameters, we achieved a comparable, yet only moderate performance, which was insufficient for robust outcome prediction in daily practice.^32^ As all tested models showed a comparable performance, we assume that these result from the inherent heterogeneity of the syndrome. However, it is worth mentioning that by using MCCV we ruled out over-optimistic model performances and ensured a robust feature importance analysis. In contrast to a single training-test set split, which can result in a randomly favourable combination and overoptimistic values, 100 splits were performed in MCCV and the mean model metrics were reported. Moreover, the primary aim of our ML approach was not to generate a clinical decision support or prediction tool, but rather to identify key proteins through the application of a feature importance analysis. The insights gained from this analysis underlined the relevance of processes that were also evident in the univariate analysis, such as blood coagulation and complement activation. Individual proteins were highlighted by their consistent appearance among the most important features within the classifiers, such as F13B, FETUB, LCAT, MB and VWF.

We evaluated the generalizability of our study by comparing it to a large cohort of patients with sepsis that was recently described by Mi and colleagues.^9^ The compared studies were quite complementary, as our cohort had a higher 30-day mortality of 38% compared to 17.1% and more severe sepsis as indicated by a median SOFA score of 9 (IQR 6–12) compared to 6 (IQR 3–8). Consistent with this, proteins related to processes associated with severe sepsis, such as liver failure, tissue damage and blood coagulation were highlighted in our study, whereas kidney injury, cardiovascular functions and coagulation were found to influence the composition of the plasma proteome in the study of Mi et al. In addition, the applied LC-MS/MS setups as well as the data acquisition and data analysis strategies were complementary, which also led to partially complementary results (meant that out of 269 proteins in the core data set of Mi et al. 176 were also covered by our analysis). This represents the current challenges in comparing data sets in the dynamically evolving filed of proteomics, as to date only few common standards exist. Nevertheless, the study by Mi et al. was clearly the most suitable for comparison due to its large number of patients and in-depth analysis. Although the number of commonly significant proteins may appear small at first glance - although this was to be expected given the complementarity mentioned above - we found a striking agreement in the relative changes in protein abundance that were associated with sepsis mortality. These observations strengthen the significance of both studies and additionally underline the relevance of proteins such as KNG1, HRG and S100A8/S100A9. However, additional investigations are needed to complete our view on the molecular changes in sepsis notably in terms of the breadth of the proteome which is assayed and kinetics of response.

Taken together, our findings enhance the understanding of plasma proteome dynamics in relation to sepsis survival and severity. We shed light on the intricate interactions between disease processes and the plasma proteome and highlight relevant biological networks. Next to well known processes such as tissue damage and coagulation disorders, we find complement activation as a major player that is poorly represented in routine diagnostics. By characterizing changes in the plasma proteome at critical stages of the disease and over time, we are providing valuable insights into the molecular mechanisms underlying sepsis, ultimately contributing to more targeted and effective therapeutic strategies.

### Limitations

Due to limitations in instrumentation and the specific protein composition of plasma, it is currently extremely challenging to measure low-abundance proteins, such as certain cytokines, using LC-MS/MS. Consequently, our observations are limited to the top 600 plasma proteins, and we cannot make statements regarding lower abundant proteins. We measured our cohort distributed over several batches which introduced additional technical variation. We performed a batch normalization and thorough quality control to account for this. Another limitation of our study is the lack of a non-septic ICU control group, which could clarify whether the identified protein regulations are specific to sepsis or also occur in other critically ill patients.

### Conclusion

Our proteomics approach revealed significant associations between changes in the plasma proteome within the first days of sepsis and mortality as well as disease severity. We identified and characterised the underlying protein networks, providing insights beyond traditional clinical assays. Key processes within the innate immune system, such as complement activation, were associated with sepsis outcome but are not typically assessed in routine diagnostics. Future investigations should focus on the role of individual proteins and their potential as diagnostic or therapeutic targets.

## Contributors

TB, LP, MA, BS and designed the study. TB, LP, MW wrote the original draft and MA, AW, LB, BS, HN, ME, TR, BK, KR, DZ, UL, DH, SFE, AZ, YM, RF, JCK revised the original draft of the manuscript. LP, TR, AW, LB, UL, DH, SFE, AZ, MA, MB, YM recruited patients and generated data. TB, MW, LP, HN, KS, YM analysed data. YM, JCK, RF provided data for validation. MA and BS supervised the study. All authors have read and approved the final version of the manuscript. TB, MW and LP accessed and verified the underlying data.

## Data sharing statement

The mass spectrometry proteomics data have been deposited to the ProteomeXchange Consortium via the PRIDE partner repository with the dataset identifier PXD055932.

## Declaration of interests

AZ received payments for grants, lectures or consulting from Baxter BioMerieux, Bayer, AM Pharma, Novartis, RenalGuard, Alexion, Baxter, Paion and Viatris. JCK received funding from Danaher Beacon. AZ has leadership or fiduciary role in IARS, DIVI and DGAI. ME is employee of Ruhr-University Bochum.
