# Supplementary material for: Mortality-associated plasma proteome dynamics in a prospective multicentre sepsis cohort
Source: eBioMedicine. 2024 Dec 15;111:105508. doi: 10.1016/j.ebiom.2024.105508 (PMC11714398; doi:10.1016/j.ebiom.2024.105508)
Supplement: Supplementary Methods, Figs. S1–S4, and Tables S1–S7 [file mmc1.docx]

# Mortality-associated Plasma Proteome Dynamics in a Prospective Multicenter Sepsis Cohort

Lars Palmowski, Maike Weber, Malte Bayer, Yuxin Mi, Karin Schork, Martin Eisenacher, Hartmuth Nowak, Tim Rahmel, Lars Bergmann, Andrea Witowski, Björn Koos, Katharina Rump, Dominik Ziehe, Ulrich Limper, Dietrich Henzler, Stefan Felix Ehrentraut, Alexander Zarbock, Roman Fischer, Julian C. Knight, Michael Adamzik, Barbara Sitek, Thilo Bracht

Supplementary Material

| **Table of Contents** | |
| --- | --- |
| 1 | Supplementary Methods |
| 2 | Supplementary Table 1: Optimized hyperparameters for the three random forest classifiers |
| 3 | Supplementary Table 2: Significantly differentially abundant proteins from 30-day survival analysis |
| 8 | Supplementary Table 3: Overview table of proteins that were significantly differentially abundant or selected as machine learning feature |
| 4 | Supplementary Table 4: Mean performances of the evaluated machine learning models. |
| 5 | Supplementary Table 5: Feature importance ranks for random forest classifiers |
| 6 | Supplementary Table 6: Numbers of survivors and non-survivors for the study of Mi et al. |
| 7 | Supplementary Table 7: Significantly differentially abundant proteins from the study of Mi et al. |
| 9 | Supplementary Figure 1: PCA analysis before and after batch normalization |
| 10 | Supplementary Figure 2: Boxplot representation before and after batch normalization |
| 11 | Supplementary Figure 3: MA plots showing the relative changes in protein intensities between day 1 and day 4 for deceased and survived patients. |
| 12 | Supplementary Figure 4: Linear regression analysis was performed for all significantly regulated proteins with the SOFA score separately for days 1 and 4 |

**Supplementary Methods**

*Proteomics sample preparation*

All chemicals were purchased from Sigma-Aldrich or Thermo Fisher Scientific unless otherwise stated. Briefly, 1 µl of plasma per sample was diluted 1:24 (v/v) in 100 mM Tris(hydroxymethyl)aminomethane pH 8.5, 10 mM Tris(2-carboxyethyl)phosphine, 40 mM 2 chloroacetamide and 1% sodium deoxycholate and incubated at 95°C for 10 min. Then, 28 µL ammonium bicarbonate (Ambic, 50 mM) and 400 µg paramagnetic Sera-Mag carboxyl magnet beads (Beads, Cytiva, GE Healthcare, Chicago, IL) were added. Acetonitrile (ACN) was added to a final concentration of 70% and the mixture was incubation for 18 min with periodic vortexing. The beads were then washed twice with 70% ethanol and once with ACN. The remaining ACN was allowed to evaporate before digestion of the proteins with 1.5 µg trypsin (SERVA Electrophoresis, Heidelberg, Germany) in 50 mM Ambic at 37 °C overnight. The resulting peptides were transferred to a new reaction tube and digestion was stopped by adding 10% trifluoroacetic acid (TFA) to a final concentration of 0.1% (v/v).

*LC-MS/MS analysis*

579 samples from 363 patients were analysed in eight batches using three different LC-MS setups. Batch S3 was measured using an Ultimate 3000 RSLCnano HPLC coupled to an Orbitrap Fusion Lumos mass spectrometer (both Thermo Scientific, Bremen, Germany). Peptides were preconcentrated on a trap column (Acclaim PepMap 100, 75 μm × 2 cm, C18, 5 μm, 100 Å) for 7 min at a flow rate of 30 μl/min using 0.1% TFA. The separation was then performed on an analytical column (Acclaim PepMap RSLC, 75 μm × 50 cm, nano Viper, C18, 5 μm, 100 Å) with a gradient of 5-30% solvent B over 38 min at a flow rate of 400 nl/min and a column temperature of 60 °C (solvent A: 0.1% formic acid (FA); solvent B: 0.1% FA, 84% ACN). The Fusion Lumos was operated in DIA mode with 21 windows between 350 and 1400 m/z. The MS1 resolution was set to 60k with an RF lens setting of 30%, a maximum injection time (MIT) of 80 ms and an automatic gain control (AGC) target of 4e5. For MS2 scans, ions were fragmented using HCD with a normalized collision energy (nCE) of 28%. The resolution was set to 30k in a m/z range of 372 to 1275. The MIT was set to 54 ms and the AGC to 375000. Batch C3 was analysed on a Vanquish Neo UHPLC coupled to an Orbitrap Exploris 240. Separation was achieved at a flow rate of 0.5 µL/min, a column temperature of 60°C and a gradient from 1% B to 30% B in the first 50 min, to 24% B in a further 2 min and finally to 45% B in 3 min. This was followed by a 14-minute wash step with alternating concentrations of 99% and 1% B. In contrast to the Ultimate 3000 RSLCnano HPLC setups, a concentration of 80% ACN in 0.1% FA was used as solvent B. The Exploris 240 was operated in DIA mode with 23 windows between 380 and 1380 m/z. The MS1 resolution was set to 120k, the RF lens to 85%, the AGC to 1e6 and the MIT to auto. For MS2 the HCD nCE was set to 30%, the scan range to 145-1450 m/z, the resolution to 30k, the AGC target to 1e6 and the MIT to 80 ms. All other batches were analysed on an Ultimate 3000 RSLCnano HPLC coupled to an Orbitrap Exploris 240. The MS settings were the same as above. For the separation of the analytes, either a 15 cm self-packed analytical column (C18, ReproSil-Pur (Dr. Maisch HPLC GmbH, Ammerbuch, Germany), 75 µm x 15 cm, 1.9 µm particle size, 120 Å pore size) with a flow rate of 400 nL/min or a DNV PepMap 1500 bar C18 analytical column (75 mm x 150 mm, 2 µm particle size, 100 Å pore size, Thermo Fisher Scientific) with a flow rate of 500 nL/min was used. The separation was carried out using a 40 min gradient of 1-35% solvent B, where 1% solvent B was maintained for the first 7 min, followed by two increasing steps to 25% solvent B for 28 min and then to 35% B for 5 min. The columns were heated to 60 °C.

*Proteomics data analysis*

A spectral library was generated from DDA data acquired using a Fusion Lumos or QExactive HF instrument as previously described (Unterberg et al., Crit Care. 2023 Oct 31;27(1):417). The respective raw data were processed using FragPipe (v.18) with default settings and searching the UniProt/SwissProt database restricted to Homo sapiens (v.2022_05). The DIA data were processed with DIA-NN (ver.1.8.1) using the spectral library and database mentioned above. Deep learning was enabled and default parameters were used, expect for heuristic protein inference was disabled and the network classifier was set to double-pass mode. All batches were processed individually and normalized by the software (RT-dependent). The resulting data sets were then imported into R and merged by the first accession of the protein groups. The protein intensities of each batch were first log2 transformed and normalized separately using the Loess method (Välikangas et al., Brief Bioinform. 2018 Jan 1;19(1):1-11.). A linear model was then calculated for each protein to estimate the batch effect for each batch. This batch effect was then subtracted from the protein intensities of the corresponding batch to reduce differences between batches. The quality of the batch normalization was assessed using box plots, principal component analysis (PCA) plots, heat maps and MA plots. Differences in protein intensities between groups (survival, sepsis and covid) were tested for statistical significance using t-test. Proteins with at least five observations per condition were considered for testing and p-values were corrected using the Benjamini-Hochberg method. Ratios of mean intensities were calculated based on delogarithmized intensities. Proteins with a pFDR value ≤ 0.05 were considered significant. Functional annotation and enrichment analyses were performed using the STRING web interface (string-db.org, v.12.0).

**Supplementary Tables**

Supplementary Table 1: Optimized hyperparameters for the three random forest classifiers.

| **Hyperparameters** | **30-day survival**  **(day 1)** | **4-day survival**  **(day 1)** | **30-day survival**  **(days 1 and 4)** |
| --- | --- | --- | --- |
| min_samples_leaf | 1 | 1 | 1 |
| min_samples_split | 2 | 2 | 2 |
| n_estimators | 100 | 100 | 600 |
| max_features | log2 | log2 | sqrt |
| max_depth | null | null | 90 |
| class_weight | balanced | balanced | balanced |
| binary threshold | 0.4 | 0.4 | 0.4 |

**Supplementary Table 2:** Significantly differentially abundant proteins from 30-day survival analysis.

**Day 1 Proteome**

| **Protein** | **Gene** | **Description** | **t-test p_FDR_** | **Patients Survival** | **Patients Death** | **Ratio of means (Death /Survival)** |
| --- | --- | --- | --- | --- | --- | --- |
| P13796 | LCP1 | Plastin-2 | 8.49E-06 | 203 | 130 | 1.41 |
| O60814 | H2BC12 | Histone H2B type 1-K | 3.34E-05 | 175 | 122 | 3.60 |
| P00338 | LDHA | L-lactate dehydrogenase A chain | 3.34E-05 | 165 | 120 | 3.26 |
| P29401 | TKT | Transketolase | 6.84E-05 | 157 | 113 | 1.64 |
| Q13790 | APOF | Apolipoprotein F | 6.84E-05 | 183 | 117 | 0.71 |
| P12259 | F5 | Coagulation factor V | 1.07E-04 | 204 | 130 | 0.83 |
| P04040 | CAT | Catalase | 1.16E-04 | 161 | 113 | 1.40 |
| P05062 | ALDOB | Fructose-bisphosphate aldolase B | 1.16E-04 | 153 | 114 | 2.98 |
| Q9UGM5 | FETUB | Fetuin-B | 1.16E-04 | 198 | 129 | 0.69 |
| P04275 | VWF | von Willebrand factor | 1.17E-04 | 204 | 130 | 1.46 |
| P0C0S8 | H2AC17 | Histone H2A type 1 | 1.17E-04 | 158 | 119 | 3.87 |
| P37837 | TALDO1 | Transaldolase | 1.38E-04 | 118 | 103 | 1.77 |
| P02144 | MB | Myoglobin | 1.90E-04 | 135 | 113 | 2.05 |
| P04196 | HRG | Histidine-rich glycoprotein | 2.94E-04 | 204 | 130 | 0.81 |
| P05160 | F13B | Coagulation factor XIII B chain | 5.60E-04 | 202 | 126 | 0.83 |
| P99999 | CYCS | Cytochrome c | 6.86E-04 | 41 | 48 | 2.24 |
| P04180 | LCAT | Phosphatidylcholine-sterol acyltransferase | 8.71E-04 | 203 | 130 | 0.86 |
| P11021 | HSPA5 | Endoplasmic reticulum chaperone BiP | 8.71E-04 | 190 | 122 | 1.48 |
| P01042 | KNG1 | Kininogen-1 | 1.16E-03 | 204 | 130 | 0.91 |
| P07339 | CTSD | Cathepsin D | 1.66E-03 | 154 | 109 | 1.29 |
| P07900 | HSP90AA1 | Heat shock protein HSP 90-alpha | 1.71E-03 | 154 | 113 | 1.71 |
| P00915 | CA1 | Carbonic anhydrase 1 | 2.32E-03 | 199 | 127 | 1.42 |
| Q9BXR6 | CFHR5 | Complement factor H-related protein 5 | 3.71E-03 | 202 | 127 | 0.80 |
| P20851 | C4BPB | C4b-binding protein beta chain | 4.97E-03 | 188 | 125 | 0.84 |
| Q16610 | ECM1 | Extracellular matrix protein 1 | 4.97E-03 | 158 | 97 | 0.85 |
| P04075 | ALDOA | Fructose-bisphosphate aldolase A | 5.27E-03 | 169 | 120 | 1.52 |
| P08263 | GSTA1 | Glutathione S-transferase A1 | 5.27E-03 | 92 | 76 | 3.96 |
| P60709 | ACTB | Actin, cytoplasmic 1 | 5.27E-03 | 204 | 130 | 1.96 |
| P08238 | HSP90AB1 | Heat shock protein HSP 90-beta | 5.98E-03 | 104 | 85 | 1.63 |
| P01024 | C3 | Complement C3 | 7.11E-03 | 204 | 130 | 0.90 |
| P63104 | YWHAZ | 14-3-3 protein zeta/delta | 7.11E-03 | 148 | 99 | 1.34 |
| P02452 | COL1A1 | Collagen alpha-1(I) chain | 8.09E-03 | 78 | 72 | 1.79 |
| P07437 | TUBB | Tubulin beta chain | 8.09E-03 | 86 | 58 | 0.68 |
| P32119 | PRDX2 | Peroxiredoxin-2 | 8.09E-03 | 202 | 129 | 1.30 |
| P62805 | H4C16 | Histone H4 | 8.09E-03 | 117 | 102 | 2.67 |
| P69905 | HBA2 | Hemoglobin subunit alpha | 8.09E-03 | 204 | 130 | 1.29 |
| O95954 | FTCD | Formimidoyltransferase-cyclodeaminase | 8.09E-03 | 25 | 34 | 2.48 |
| P01717 | IGLV3-25 | Immunoglobulin lambda variable 3-25 | 8.09E-03 | 86 | 40 | 0.52 |
| P26927 | MST1 | Hepatocyte growth factor-like protein | 8.74E-03 | 188 | 124 | 0.84 |
| P24158 | PRTN3 | Myeloblastin | 9.17E-03 | 144 | 108 | 1.42 |
| P00739 | HPR | Haptoglobin-related protein | 9.29E-03 | 204 | 129 | 0.84 |
| P01782 | IGHV3-9 | Immunoglobulin heavy variable 3-9 | 9.29E-03 | 115 | 59 | 0.74 |
| P04179 | SOD2 | Superoxide dismutase [Mn], mitochondrial | 9.29E-03 | 127 | 90 | 1.85 |
| P04406 | GAPDH | Glyceraldehyde-3-phosphate dehydrogenase | 9.29E-03 | 145 | 109 | 2.23 |
| P36222 | CHI3L1 | Chitinase-3-like protein 1 | 9.29E-03 | 140 | 106 | 1.54 |
| P04070 | PROC | Vitamin K-dependent protein C | 1.13E-02 | 195 | 121 | 0.90 |
| O75882 | ATRN | Attractin | 1.16E-02 | 203 | 130 | 0.89 |
| Q13228 | SELENBP1 | Methanethiol oxidase | 1.19E-02 | 104 | 86 | 1.23 |
| P36980 | CFHR2 | Complement factor H-related protein 2 | 1.19E-02 | 86 | 45 | 0.72 |
| P19971 | TYMP | Thymidine phosphorylase | 1.24E-02 | 68 | 80 | 1.54 |
| P05452 | CLEC3B | Tetranectin | 1.25E-02 | 180 | 105 | 0.85 |
| P21549 | AGXT | Alanine--glyoxylate aminotransferase | 1.25E-02 | 102 | 79 | 2.13 |
| Q9Y279 | VSIG4 | V-set and immunoglobulin domain-containing protein 4 | 1.25E-02 | 144 | 102 | 1.41 |
| P04004 | VTN | Vitronectin | 1.27E-02 | 204 | 130 | 0.89 |
| Q92496 | CFHR4 | Complement factor H-related protein 4 | 1.53E-02 | 156 | 104 | 0.77 |
| P09210 | GSTA2 | Glutathione S-transferase A2 | 1.66E-02 | 6 | 19 | 8.61 |
| P78417 | GSTO1 | Glutathione S-transferase omega-1 | 1.76E-02 | 166 | 115 | 1.81 |
| P13473 | LAMP2 | Lysosome-associated membrane glycoprotein 2 | 1.84E-02 | 111 | 51 | 0.72 |
| Q7Z4W1 | DCXR | L-xylulose reductase | 1.90E-02 | 27 | 43 | 2.16 |
| A0A087WSZ0 | IGKV1D-8 | Immunoglobulin kappa variable 1D-8 | 1.95E-02 | 196 | 120 | 0.87 |
| P68871 | HBB | Hemoglobin subunit beta | 2.07E-02 | 204 | 130 | 1.30 |
| P12955 | PEPD | Xaa-Pro dipeptidase | 2.17E-02 | 138 | 104 | 1.28 |
| Q16851 | UGP2 | UTP--glucose-1-phosphate uridylyltransferase | 2.17E-02 | 91 | 97 | 1.92 |
| P08833 | IGFBP1 | Insulin-like growth factor-binding protein 1 | 2.34E-02 | 68 | 76 | 1.48 |
| P16930 | FAH | Fumarylacetoacetase | 2.42E-02 | 56 | 68 | 1.86 |
| P00488 | F13A1 | Coagulation factor XIII A chain | 2.44E-02 | 201 | 127 | 0.87 |
| P68431 | H3C12 | Histone H3.1 | 2.44E-02 | 79 | 78 | 2.73 |
| P55056 | APOC4 | Apolipoprotein C-IV | 2.50E-02 | 191 | 115 | 0.83 |
| P49913 | CAMP | Cathelicidin antimicrobial peptide | 3.06E-02 | 158 | 89 | 0.75 |
| P07237 | P4HB | Protein disulfide-isomerase | 3.09E-02 | 117 | 91 | 1.55 |
| P26038 | MSN | Moesin | 3.20E-02 | 130 | 102 | 1.29 |
| O43866 | CD5L | CD5 antigen-like | 3.43E-02 | 203 | 129 | 1.19 |
| P08514 | ITGA2B | Integrin alpha-IIb | 3.43E-02 | 136 | 80 | 0.76 |
| P40925 | MDH1 | Malate dehydrogenase, cytoplasmic | 3.43E-02 | 36 | 49 | 2.04 |
| P01602 | IGKV1-5 | Immunoglobulin kappa variable 1-5 | 3.62E-02 | 173 | 111 | 0.92 |
| Q00266 | MAT1A | S-adenosylmethionine synthase isoform type-1 | 3.62E-02 | 20 | 32 | 2.60 |
| O00151 | PDLIM1 | PDZ and LIM domain protein 1 | 3.62E-02 | 44 | 31 | 0.72 |
| Q15942 | ZYX | Zyxin | 3.63E-02 | 104 | 68 | 0.75 |
| P14618 | PKM | Pyruvate kinase PKM | 3.79E-02 | 23 | 27 | 0.71 |
| P40926 | MDH2 | Malate dehydrogenase, mitochondrial | 4.02E-02 | 11 | 19 | 1.96 |
| P0C0L5 | C4B_2 | Complement C4-B | 4.17E-02 | 204 | 130 | 0.90 |
| A0A0B4J1Y9 | IGHV3-72 | Immunoglobulin heavy variable 3-72 | 4.24E-02 | 180 | 108 | 0.86 |
| P05109 | S100A8 | Protein S100-A8 | 4.25E-02 | 200 | 128 | 1.57 |
| P14550 | AKR1A1 | Aldo-keto reductase family 1 member A1 | 4.57E-02 | 25 | 37 | 1.73 |
| P07451 | CA3 | Carbonic anhydrase 3 | 4.94E-02 | 33 | 55 | 1.18 |
| P59998 | ARPC4 | Actin-related protein 2/3 complex subunit 4 | 4.94E-02 | 13 | 18 | 0.70 |
| P05106 | ITGB3 | Integrin beta-3 | 4.98E-02 | 29 | 18 | 0.61 |

**Day 4 Proteome**

| **Protein** | **Gene** | **Description** | **t-test p_FDR_** | **Patients Survival** | **Patients Death** | **Ratio of means (Death /Survival)** |
| --- | --- | --- | --- | --- | --- | --- |
| P04075 | ALDOA | Fructose-bisphosphate aldolase A | 5.27E-07 | 142 | 93 | 1.60 |
| P02144 | MB | Myoglobin | 1.09E-06 | 96 | 82 | 7.26 |
| P37837 | TALDO1 | Transaldolase | 2.01E-05 | 93 | 76 | 2.01 |
| P00338 | LDHA | L-lactate dehydrogenase A chain | 3.22E-05 | 120 | 84 | 3.62 |
| O60814 | H2BC12 | Histone H2B type 1-K | 4.03E-05 | 131 | 90 | 4.62 |
| P0C0S8 | H2AC17 | Histone H2A type 1 | 4.03E-05 | 124 | 87 | 4.44 |
| P05160 | F13B | Coagulation factor XIII B chain | 2.35E-04 | 151 | 91 | 0.79 |
| P04196 | HRG | Histidine-rich glycoprotein | 3.72E-04 | 152 | 93 | 0.79 |
| P04180 | LCAT | Phosphatidylcholine-sterol acyltransferase | 5.60E-04 | 150 | 89 | 0.81 |
| P20742 | PZP | Pregnancy zone protein | 1.06E-03 | 132 | 82 | 0.66 |
| P60709 | ACTB | Actin, cytoplasmic 1 | 1.27E-03 | 152 | 93 | 2.28 |
| P04070 | PROC | Vitamin K-dependent protein C | 1.47E-03 | 145 | 83 | 0.79 |
| P00747 | PLG | Plasminogen | 2.81E-03 | 152 | 93 | 0.86 |
| P04040 | CAT | Catalase | 2.91E-03 | 114 | 82 | 1.58 |
| P07900 | HSP90AA1 | Heat shock protein HSP 90-alpha | 2.91E-03 | 114 | 82 | 1.93 |
| P36222 | CHI3L1 | Chitinase-3-like protein 1 | 2.91E-03 | 79 | 68 | 2.00 |
| P62805 | H4C16 | Histone H4 | 2.91E-03 | 93 | 74 | 3.14 |
| P29401 | TKT | Transketolase | 3.10E-03 | 99 | 80 | 1.74 |
| Q15848 | ADIPOQ | Adiponectin | 3.41E-03 | 95 | 60 | 0.71 |
| P12259 | F5 | Coagulation factor V | 3.75E-03 | 152 | 93 | 0.86 |
| P13796 | LCP1 | Plastin-2 | 3.75E-03 | 151 | 93 | 1.51 |
| P04179 | SOD2 | Superoxide dismutase [Mn], mitochondrial | 4.15E-03 | 96 | 65 | 2.85 |
| P09467 | FBP1 | Fructose-1,6-bisphosphatase 1 | 5.49E-03 | 25 | 31 | 2.61 |
| P05109 | S100A8 | Protein S100-A8 | 5.92E-03 | 151 | 92 | 1.49 |
| P68032 | ACTC1 | Actin, alpha cardiac muscle 1 | 5.92E-03 | 126 | 81 | 1.59 |
| P05154 | SERPINA5 | Plasma serine protease inhibitor | 6.64E-03 | 152 | 91 | 0.80 |
| P00450 | CP | Ceruloplasmin | 6.82E-03 | 152 | 93 | 1.11 |
| P04004 | VTN | Vitronectin | 6.82E-03 | 152 | 93 | 0.86 |
| P15169 | CPN1 | Carboxypeptidase N catalytic chain | 6.82E-03 | 152 | 93 | 1.17 |
| P01034 | CST3 | Cystatin-C | 6.90E-03 | 151 | 93 | 1.27 |
| Q06033 | ITIH3 | Inter-alpha-trypsin inhibitor heavy chain H3 | 7.40E-03 | 152 | 93 | 1.15 |
| P04275 | VWF | von Willebrand factor | 7.43E-03 | 152 | 93 | 1.38 |
| P05062 | ALDOB | Fructose-bisphosphate aldolase B | 7.43E-03 | 118 | 82 | 3.06 |
| P14625 | HSP90B1 | Endoplasmin | 7.43E-03 | 112 | 78 | 1.35 |
| Q03154 | ACY1 | Aminoacylase-1 | 7.43E-03 | 35 | 45 | 2.13 |
| P01019 | AGT | Angiotensinogen | 7.53E-03 | 152 | 93 | 1.23 |
| P02790 | HPX | Hemopexin | 7.57E-03 | 152 | 93 | 0.85 |
| Q9UGM5 | FETUB | Fetuin-B | 7.70E-03 | 147 | 86 | 0.80 |
| Q14520 | HABP2 | Hyaluronan-binding protein 2 | 8.01E-03 | 151 | 89 | 0.84 |
| A0A0B4J1Y8 | IGLV9-49 | Immunoglobulin lambda variable 9-49 | 8.71E-03 | 135 | 82 | 0.68 |
| P68431 | H3C12 | Histone H3.1 | 8.71E-03 | 52 | 52 | 2.69 |
| P05452 | CLEC3B | Tetranectin | 8.82E-03 | 134 | 73 | 0.80 |
| A0A0B4J1Y9 | IGHV3-72 | Immunoglobulin heavy variable 3-72 | 8.99E-03 | 137 | 79 | 0.78 |
| O75882 | ATRN | Attractin | 8.99E-03 | 152 | 92 | 0.88 |
| P07195 | LDHB | L-lactate dehydrogenase B chain | 9.10E-03 | 149 | 93 | 1.38 |
| P07339 | CTSD | Cathepsin D | 1.02E-02 | 106 | 73 | 1.33 |
| P14618 | PKM | Pyruvate kinase PKM | 1.16E-02 | 17 | 21 | 0.57 |
| P15259 | PGAM2 | Phosphoglycerate mutase 2 | 1.16E-02 | 48 | 48 | 1.79 |
| P02649 | APOE | Apolipoprotein E | 1.21E-02 | 152 | 93 | 1.22 |
| P05546 | SERPIND1 | Heparin cofactor 2 | 1.25E-02 | 152 | 93 | 0.86 |
| P07237 | P4HB | Protein disulfide-isomerase | 1.38E-02 | 81 | 65 | 1.49 |
| P10909 | CLU | Clusterin | 1.38E-02 | 152 | 93 | 0.90 |
| P07148 | FABP1 | Fatty acid-binding protein, liver | 1.40E-02 | 10 | 23 | 4.73 |
| P49913 | CAMP | Cathelicidin antimicrobial peptide | 1.43E-02 | 117 | 69 | 0.74 |
| P49908 | SELENOP | Selenoprotein P | 1.61E-02 | 152 | 93 | 0.87 |
| P01008 | SERPINC1 | Antithrombin-III | 1.68E-02 | 152 | 93 | 0.91 |
| P28066 | PSMA5 | Proteasome subunit alpha type-5 | 1.68E-02 | 56 | 62 | 1.64 |
| Q92496 | CFHR4 | Complement factor H-related protein 4 | 1.75E-02 | 122 | 67 | 0.76 |
| P78417 | GSTO1 | Glutathione S-transferase omega-1 | 1.99E-02 | 121 | 82 | 1.58 |
| P01782 | IGHV3-9 | Immunoglobulin heavy variable 3-9 | 2.09E-02 | 100 | 41 | 0.75 |
| P08185 | SERPINA6 | Corticosteroid-binding globulin | 2.09E-02 | 152 | 93 | 0.90 |
| P26038 | MSN | Moesin | 2.10E-02 | 84 | 72 | 1.47 |
| P05156 | CFI | Complement factor I | 2.15E-02 | 152 | 93 | 0.88 |
| P06702 | S100A9 | Protein S100-A9 | 2.15E-02 | 150 | 92 | 1.40 |
| Q9NZP8 | C1RL | Complement C1r subcomponent-like protein | 2.16E-02 | 152 | 93 | 0.90 |
| P01764 | IGHV3-23 | Immunoglobulin heavy variable 3-23 | 2.38E-02 | 149 | 90 | 0.75 |
| P37802 | TAGLN2 | Transgelin-2 | 2.38E-02 | 41 | 28 | 0.70 |
| P00739 | HPR | Haptoglobin-related protein | 2.39E-02 | 152 | 92 | 0.84 |
| P10643 | C7 | Complement component C7 | 2.39E-02 | 152 | 93 | 1.20 |
| Q16610 | ECM1 | Extracellular matrix protein 1 | 2.39E-02 | 122 | 68 | 0.86 |
| P02741 | CRP | C-reactive protein | 2.42E-02 | 152 | 93 | 1.18 |
| P12955 | PEPD | Xaa-Pro dipeptidase | 2.42E-02 | 98 | 72 | 1.42 |
| P20851 | C4BPB | C4b-binding protein beta chain | 2.48E-02 | 145 | 84 | 0.86 |
| Q03591 | CFHR1 | Complement factor H-related protein 1 | 2.63E-02 | 152 | 91 | 0.82 |
| P26927 | MST1 | Hepatocyte growth factor-like protein | 2.71E-02 | 146 | 84 | 0.84 |
| P36980 | CFHR2 | Complement factor H-related protein 2 | 2.71E-02 | 66 | 32 | 0.72 |
| P16930 | FAH | Fumarylacetoacetase | 2.89E-02 | 44 | 55 | 2.02 |
| P69905 | HBA2 | Hemoglobin subunit alpha | 3.26E-02 | 152 | 93 | 1.71 |
| P03951 | F11 | Coagulation factor XI | 3.35E-02 | 149 | 90 | 0.88 |
| P21549 | AGXT | Alanine--glyoxylate aminotransferase | 3.55E-02 | 87 | 55 | 2.06 |
| P01024 | C3 | Complement C3 | 3.64E-02 | 152 | 93 | 0.90 |
| P02766 | TTR | Transthyretin | 3.65E-02 | 152 | 93 | 0.82 |
| P06733 | ENO1 | Alpha-enolase | 3.65E-02 | 88 | 66 | 1.36 |
| P68104 | EEF1A1 | Elongation factor 1-alpha 1 | 3.65E-02 | 63 | 66 | 1.38 |
| P00748 | F12 | Coagulation factor XII | 3.67E-02 | 152 | 93 | 0.87 |
| P11021 | HSPA5 | Endoplasmic reticulum chaperone BiP | 3.72E-02 | 145 | 90 | 1.60 |
| P01042 | KNG1 | Kininogen-1 | 3.75E-02 | 152 | 93 | 0.93 |
| P08238 | HSP90AB1 | Heat shock protein HSP 90-beta | 3.89E-02 | 62 | 60 | 1.45 |
| Q9Y5Y7 | LYVE1 | Lymphatic vessel endothelial hyaluronic acid receptor 1 | 4.14E-02 | 81 | 48 | 0.81 |
| P52209 | PGD | 6-phosphogluconate dehydrogenase, decarboxylating | 4.22E-02 | 24 | 34 | 1.43 |
| P49720 | PSMB3 | Proteasome subunit beta type-3 | 4.35E-02 | 9 | 15 | 0.38 |
| P63104 | YWHAZ | 14-3-3 protein zeta/delta | 4.41E-02 | 95 | 73 | 1.30 |
| P11226 | MBL2 | Mannose-binding protein C | 4.48E-02 | 121 | 80 | 0.81 |
| P25787 | PSMA2 | Proteasome subunit alpha type-2 | 4.49E-02 | 24 | 36 | 1.61 |
| P13473 | LAMP2 | Lysosome-associated membrane glycoprotein 2 | 4.60E-02 | 75 | 35 | 0.74 |

**Day 4 / Day 1 Ratios**

| **Protein** | **Gene** | **Description** | **t-test p_FDR_** | **Patients Survival** | **Patients Death** | **Mean(Ratio) Survival** | **Mean(Ratio) Death** |
| --- | --- | --- | --- | --- | --- | --- | --- |
| Q14520 | HABP2 | Hyaluronan-binding protein 2 | 2.18E-05 | 260 | 152 | 1.30 | 1.02 |
| P35858 | IGFALS | Insulin-like growth factor-binding protein complex acid labile subunit | 3.12E-04 | 262 | 166 | 1.10 | 0.85 |
| P02790 | HPX | Hemopexin | 4.56E-04 | 264 | 168 | 0.97 | 0.85 |
| P02792 | FTL | Ferritin light chain | 4.56E-04 | 48 | 36 | 1.16 | 5.41 |
| P03952 | KLKB1 | Plasma kallikrein | 4.56E-04 | 264 | 168 | 1.06 | 0.94 |
| P04180 | LCAT | Phosphatidylcholine-sterol acyltransferase | 4.56E-04 | 258 | 160 | 1.09 | 0.95 |
| Q15848 | ADIPOQ | Adiponectin | 4.56E-04 | 154 | 96 | 1.21 | 0.91 |
| P01019 | AGT | Angiotensinogen | 4.66E-04 | 264 | 168 | 1.11 | 1.40 |
| P04070 | PROC | Vitamin K-dependent protein C | 4.66E-04 | 244 | 146 | 1.15 | 1.01 |
| Q9NZP8 | C1RL | Complement C1r subcomponent-like protein | 4.66E-04 | 264 | 168 | 1.11 | 0.98 |
| P02750 | LRG1 | Leucine-rich alpha-2-glycoprotein | 1.63E-03 | 264 | 168 | 0.95 | 1.14 |
| P03951 | F11 | Coagulation factor XI | 1.67E-03 | 258 | 162 | 1.04 | 0.95 |
| P00450 | CP | Ceruloplasmin | 1.97E-03 | 264 | 168 | 0.99 | 1.09 |
| P05160 | F13B | Coagulation factor XIII B chain | 3.32E-03 | 260 | 160 | 1.22 | 1.10 |
| P04075 | ALDOA | Fructose-bisphosphate aldolase A | 3.41E-03 | 210 | 154 | 1.12 | 1.55 |
| P49908 | SELENOP | Selenoprotein P | 3.41E-03 | 264 | 168 | 1.24 | 1.11 |
| P08253 | MMP2 | 72 kDa type IV collagenase | 3.41E-03 | 20 | 16 | 2.05 | 0.87 |
| P04211 | IGLV7-43 | Immunoglobulin lambda variable 7-43 | 3.99E-03 | 264 | 168 | 1.05 | 1.25 |
| P06702 | S100A9 | Protein S100-A9 | 5.05E-03 | 260 | 166 | 1.07 | 1.81 |
| P06744 | GPI | Glucose-6-phosphate isomerase | 5.55E-03 | 22 | 32 | 0.90 | 2.10 |
| Q93088 | BHMT | Betaine--homocysteine S-methyltransferase 1 | 6.04E-03 | 18 | 32 | 0.49 | 12.03 |
| P00338 | LDHA | L-lactate dehydrogenase A chain | 7.81E-03 | 186 | 138 | 1.02 | 3.17 |
| P01764 | IGHV3-23 | Immunoglobulin heavy variable 3-23 | 7.81E-03 | 256 | 158 | 1.22 | 1.03 |
| P04278 | SHBG | Sex hormone-binding globulin | 7.81E-03 | 230 | 148 | 1.29 | 1.06 |
| P36955 | SERPINF1 | Pigment epithelium-derived factor | 7.81E-03 | 264 | 168 | 0.99 | 1.14 |
| P40261 | NNMT | Nicotinamide N-methyltransferase | 8.03E-03 | 10 | 12 | 0.71 | 2.68 |
| P00995 | SPINK1 | Serine protease inhibitor Kazal-type 1 | 8.03E-03 | 26 | 20 | 1.05 | 0.63 |
| P05546 | SERPIND1 | Heparin cofactor 2 | 8.19E-03 | 264 | 168 | 1.10 | 1.01 |
| P01876 | IGHA1 | Immunoglobulin heavy constant alpha 1 | 8.56E-03 | 264 | 168 | 1.10 | 1.27 |
| P02741 | CRP | C-reactive protein | 1.01E-02 | 264 | 168 | 1.89 | 1.15 |
| P00734 | F2 | Prothrombin | 1.04E-02 | 264 | 168 | 1.03 | 0.95 |
| P11226 | MBL2 | Mannose-binding protein C | 1.04E-02 | 196 | 136 | 1.28 | 1.00 |
| P27169 | PON1 | Serum paraoxonase/arylesterase 1 | 1.04E-02 | 264 | 168 | 0.95 | 0.87 |
| P04004 | VTN | Vitronectin | 1.06E-02 | 264 | 168 | 1.04 | 0.99 |
| P60709 | ACTB | Actin, cytoplasmic 1 | 1.31E-02 | 264 | 168 | 1.11 | 2.57 |
| P0C0S8 | H2AC17 | Histone H2A type 1 | 1.33E-02 | 184 | 146 | 1.81 | 6.61 |
| P29622 | SERPINA4 | Kallistatin | 1.34E-02 | 264 | 168 | 1.23 | 1.12 |
| P00367 | GLUD1 | Glutamate dehydrogenase 1, mitochondrial | 1.48E-02 | 6 | 24 | 0.36 | 2.82 |
| P68032 | ACTC1 | Actin, alpha cardiac muscle 1 | 1.50E-02 | 212 | 132 | 1.19 | 2.90 |
| P00738 | HP | Haptoglobin | 1.51E-02 | 264 | 168 | 1.36 | 1.25 |
| O60814 | H2BC12 | Histone H2B type 1-K | 1.58E-02 | 208 | 152 | 1.77 | 6.41 |
| P18428 | LBP | Lipopolysaccharide-binding protein | 1.73E-02 | 264 | 168 | 0.79 | 0.99 |
| P01008 | SERPINC1 | Antithrombin-III | 1.97E-02 | 264 | 168 | 1.03 | 0.97 |
| P27797 | CALR | Calreticulin | 2.06E-02 | 34 | 30 | 1.31 | 0.89 |
| P00747 | PLG | Plasminogen | 2.09E-02 | 264 | 168 | 1.10 | 1.03 |
| P15259 | PGAM2 | Phosphoglycerate mutase 2 | 2.38E-02 | 62 | 48 | 1.03 | 1.74 |
| P26038 | MSN | Moesin | 2.57E-02 | 120 | 112 | 0.89 | 1.29 |
| O95810 | CAVIN2 | Caveolae-associated protein 2 | 2.57E-02 | 12 | 6 | 0.92 | 0.54 |
| P36222 | CHI3L1 | Chitinase-3-like protein 1 | 2.57E-02 | 118 | 102 | 0.69 | 1.55 |
| Q03591 | CFHR1 | Complement factor H-related protein 1 | 2.58E-02 | 264 | 162 | 1.18 | 1.05 |
| A0A0C4DH34 | IGHV4-28 | Immunoglobulin heavy variable 4-28 | 2.60E-02 | 88 | 52 | 1.86 | 1.16 |
| Q9Y5Y7 | LYVE1 | Lymphatic vessel endothelial hyaluronic acid receptor 1 | 2.60E-02 | 128 | 74 | 1.29 | 1.09 |
| P24821 | TNC | Tenascin | 2.69E-02 | 100 | 74 | 0.95 | 1.62 |
| P05109 | S100A8 | Protein S100-A8 | 3.70E-02 | 256 | 164 | 1.22 | 1.75 |
| P04003 | C4BPA | C4b-binding protein alpha chain | 3.87E-02 | 264 | 168 | 1.00 | 0.94 |
| P37837 | TALDO1 | Transaldolase | 3.87E-02 | 116 | 116 | 1.03 | 1.41 |
| Q04756 | HGFAC | Hepatocyte growth factor activator | 3.87E-02 | 232 | 136 | 1.07 | 0.95 |
| Q06830 | PRDX1 | Peroxiredoxin-1 | 3.87E-02 | 58 | 58 | 1.03 | 1.37 |
| Q96IY4 | CPB2 | Carboxypeptidase B2 | 3.87E-02 | 264 | 168 | 1.17 | 1.08 |
| P0C0L5 | C4B_2 | Complement C4-B | 4.37E-02 | 264 | 168 | 0.98 | 0.91 |
| P04196 | HRG | Histidine-rich glycoprotein | 4.45E-02 | 264 | 168 | 1.14 | 1.03 |
| P02656 | APOC3 | Apolipoprotein C-III | 4.89E-02 | 264 | 168 | 1.42 | 1.38 |
| Q96PD5 | PGLYRP2 | N-acetylmuramoyl-L-alanine amidase | 4.95E-02 | 264 | 168 | 1.08 | 1.02 |

**Supplementary Table 3:** Overview table of proteins that were significantly differentially abundant or selected as machine learning feature.

|  |  | **Univariate Statistics** | | | | | | **Machine Learning** | | |
| --- | --- | --- | --- | --- | --- | --- | --- | --- | --- | --- |
|  |  | **Day 1** | | **Day 4** | | **Day 4 / Day 1 ratios** | | **30-day survival** | **4-day survival** | **30-day-survival (days 1 and 4)** |
| **Protein^1^** | **Gene** | **p_FDR_ value** | **RoM^2^** | **p_FDR_ value** | **RoM^2^** | **p_FDR_ value** | **Difference^3^** | **Median rank^4^** | **Median rank^4^** | **Median**  **rank^4^** |
| A0A0B4J1Y9 | IGHV3-72 | 4.24E-02 | 0.86 | 8.99E-03 | 0.78 |  |  |  |  |  |
| O43866 | CD5L | 3.43E-02 | 1.19 |  |  |  |  | 84 |  |  |
| O60814 | H2BC12 | 3.34E-05 | 3.60 | 4.03E-05 | 4.62 | 1.58E-02 | 4.64 |  |  |  |
| O75882 | ATRN | 1.16E-02 | 0.89 | 8.99E-03 | 0.88 |  |  |  |  | 78 |
| P00338 | LDHA | 3.34E-05 | 3.26 | 3.22E-05 | 3.62 | 7.81E-03 | 2.15 | 8 |  | 7.5, 75 |
| P00739 | HPR | 9.29E-03 | 0.84 | 2.39E-02 | 0.84 |  |  |  |  | 190 |
| P01024 | C3 | 7.11E-03 | 0.90 | 3.64E-02 | 0.90 |  |  | 88 | 18.5 |  |
| P01042 | KNG1 | 1.16E-03 | 0.91 | 3.75E-02 | 0.93 |  |  | 25 | 116 | 95.5 |
| P01782 | IGHV3-9 | 9.29E-03 | 0.74 | 2.09E-02 | 0.75 |  |  |  |  |  |
| P02144 | MB | 1.90E-04 | 2.05 | 1.09E-06 | 7.26 |  |  | 3 | 29.5 | 0, 19 |
| P04004 | VTN | 1.27E-02 | 0.89 | 6.82E-03 | 0.86 | 1.06E-02 | -0.05 | 101.5 |  |  |
| P04040 | CAT | 1.16E-04 | 1.40 | 2.91E-03 | 1.58 |  |  | 10 |  | 47 |
| P04070 | PROC | 1.13E-02 | 0.90 | 1.47E-03 | 0.79 | 4.66E-04 | -0.14 |  | 35.5 | 51.5 |
| P04075 | ALDOA | 5.27E-03 | 1.52 | 5.27E-07 | 1.60 | 3.41E-03 | 0.44 |  |  | 2 |
| P04179 | SOD2 | 9.29E-03 | 1.85 | 4.15E-03 | 2.85 |  |  |  |  |  |
| P04180 | LCAT | 8.71E-04 | 0.86 | 5.60E-04 | 0.81 | 4.56E-04 | -0.14 | 23.5 | 11 | 33.5 |
| P04196 | HRG | 2.94E-04 | 0.81 | 3.72E-04 | 0.79 | 4.45E-02 | -0.11 | 30.5 | 130.5 | 9 |
| P04275 | VWF | 1.17E-04 | 1.46 | 7.43E-03 | 1.38 |  |  | 20 | 33 | 56.5 |
| P05062 | ALDOB | 1.16E-04 | 2.98 | 7.43E-03 | 3.06 |  |  |  |  | 124 |
| P05109 | S100A8 | 4.25E-02 | 1.57 | 5.92E-03 | 1.49 | 3.70E-02 | 0.52 |  |  |  |
| P05160 | F13B | 5.60E-04 | 0.83 | 2.35E-04 | 0.79 | 3.32E-03 | -0.12 | 24.5 | 11.5 | 8 |
| P05452 | CLEC3B | 1.25E-02 | 0.85 | 8.82E-03 | 0.80 |  |  | 68.5 |  | 78.5 |
| P07237 | P4HB | 3.09E-02 | 1.55 | 1.38E-02 | 1.49 |  |  |  |  |  |
| P07900 | HSP90AA1 | 1.71E-03 | 1.71 | 2.91E-03 | 1.93 |  |  | 29 |  | 88 |
| P08238 | HSP90AB1 | 5.98E-03 | 1.63 | 3.89E-02 | 1.45 |  |  |  |  |  |
| P0C0L5 | C4B_2 | 4.17E-02 | 0.90 |  |  | 4.37E-02 | -0.07 |  |  |  |
| P0C0S8 | H2AC17 | 1.17E-04 | 3.87 | 4.03E-05 | 4.44 | 1.33E-02 | 4.79 |  |  |  |
| P11021 | HSPA5 | 8.71E-04 | 1.48 | 3.72E-02 | 1.60 |  |  | 21 |  | 30.5 |
| P12259 | F5 | 1.07E-04 | 0.83 | 3.75E-03 | 0.86 |  |  | 5 | 4 | 64.5, 89 |
| P12955 | PEPD | 2.17E-02 | 1.28 | 2.42E-02 | 1.42 |  |  |  |  |  |
| P13796 | LCP1 | 8.49E-06 | 1.41 | 3.75E-03 | 1.51 |  |  | 3 |  | 33,93 |
| P14618 | PKM | 3.79E-02 | 0.71 | 1.16E-02 | 0.57 |  |  |  |  |  |
| P16930 | FAH | 2.42E-02 | 1.86 | 2.89E-02 | 2.02 |  |  |  |  |  |
| P20851 | C4BPB | 4.97E-03 | 0.84 | 2.48E-02 | 0.86 |  |  |  |  |  |
| P21549 | AGXT | 1.25E-02 | 2.13 | 3.55E-02 | 2.06 |  |  |  |  |  |
| P26038 | MSN | 3.20E-02 | 1.29 | 2.10E-02 | 1.47 | 2.57E-02 | 0.40 |  |  |  |
| P26927 | MST1 | 8.74E-03 | 0.84 | 2.71E-02 | 0.84 |  |  |  | 9 |  |
| P29401 | TKT | 6.84E-05 | 1.64 | 3.10E-03 | 1.74 |  |  | 13 |  | 63.5, 73 |
| P32119 | PRDX2 | 8.09E-03 | 1.30 |  |  |  |  | 50.5 |  |  |
| P36222 | CHI3L1 | 9.29E-03 | 1.54 | 2.91E-03 | 2.00 | 2.57E-02 | 0.86 |  |  |  |
| P37837 | TALDO1 | 1.38E-04 | 1.77 | 2.01E-05 | 2.01 | 3.87E-02 | 0.38 |  |  |  |
| P55056 | APOC4 | 2.50E-02 | 0.83 |  |  |  |  |  | 18 |  |
| P60709 | ACTB | 5.27E-03 | 1.96 | 1.27E-03 | 2.28 | 1.31E-02 | 1.46 | 40 | 16.5 |  |
| P62805 | H4C16 | 8.09E-03 | 2.67 | 2.91E-03 | 3.14 |  |  |  |  |  |
| P63104 | YWHAZ | 7.11E-03 | 1.34 | 4.41E-02 | 1.30 |  |  |  |  |  |
| P68431 | H3C12 | 2.44E-02 | 2.73 | 8.71E-03 | 2.69 |  |  |  |  |  |
| P68871 | HBB | 2.07E-02 | 1.30 |  |  |  |  | 44.5 | 140.5 |  |
| P69905 | HBA2 | 8.09E-03 | 1.29 | 3.26E-02 | 1.71 |  |  | 37 |  |  |
| P78417 | GSTO1 | 1.76E-02 | 1.81 | 1.99E-02 | 1.58 |  |  |  |  |  |
| Q13790 | APOF | 6.84E-05 | 0.71 |  |  |  |  | 9.5 | 92.5 |  |
| Q16610 | ECM1 | 4.97E-03 | 0.85 | 2.39E-02 | 0.86 |  |  |  |  |  |
| Q92496 | CFHR4 | 1.53E-02 | 0.77 | 1.75E-02 | 0.76 |  |  |  |  |  |
| Q9UGM5 | FETUB | 1.16E-04 | 0.69 | 7.70E-03 | 0.80 |  |  | 11 | 45.5 | 24.5, 41 |
| P07339 | CTSD | 1.66E-03 | 1.29 | 1.02E-02 | 1.33 |  |  | 25 |  |  |
| P49913 | CAMP | 3.06E-02 | 0.75 | 1.43E-02 | 0.74 |  |  |  |  |  |
| Q9Y279 | VSIG4 | 1.25E-02 | 1.41 |  |  |  |  | 64 |  |  |
| P13473 | LAMP2 | 1.84E-02 | 0.72 | 4.60E-02 | 0.74 |  |  |  |  |  |
| P36980 | CFHR2 | 1.19E-02 | 0.72 | 2.71E-02 | 0.72 |  |  |  |  |  |
| P00450 | CP |  |  | 6.82E-03 | 1.11 | 1.97E-03 | 0.09 |  | 129 | 31.5 |
| P00747 | PLG |  |  | 2.81E-03 | 0.86 | 2.09E-02 | -0.07 | 80 |  |  |
| P01008 | SERPINC1 |  |  | 1.68E-02 | 0.91 | 1.97E-02 | -0.06 |  |  |  |
| P01019 | AGT |  |  | 7.53E-03 | 1.23 | 4.66E-04 | 0.29 |  |  |  |
| P01034 | CST3 |  |  | 6.90E-03 | 1.27 |  |  |  | 52 |  |
| P01764 | IGHV3-23 |  |  | 2.38E-02 | 0.75 | 7.81E-03 | -0.19 |  |  |  |
| P02741 | CRP |  |  | 2.42E-02 | 1.18 | 1.01E-02 | -0.73 |  |  |  |
| P02790 | HPX |  |  | 7.57E-03 | 0.85 | 4.56E-04 | -0.12 |  |  |  |
| P03951 | F11 |  |  | 3.35E-02 | 0.88 | 1.67E-03 | -0.10 |  |  | 33 |
| P05546 | SERPIND1 |  |  | 1.25E-02 | 0.86 | 8.19E-03 | -0.08 |  |  |  |
| P06702 | S100A9 |  |  | 2.15E-02 | 1.40 | 5.05E-03 | 0.74 |  |  |  |
| P08185 | SERPINA6 |  |  | 2.09E-02 | 0.90 |  |  |  |  | 52.5 |
| P10909 | CLU |  |  | 1.38E-02 | 0.90 |  |  |  | 106 |  |
| P11226 | MBL2 |  |  | 4.48E-02 | 0.81 | 1.04E-02 | -0.28 |  |  |  |
| P15259 | PGAM2 |  |  | 1.16E-02 | 1.79 | 2.38E-02 | 0.70 |  |  |  |
| P49908 | SELENOP |  |  | 1.61E-02 | 0.87 | 3.41E-03 | -0.13 |  |  |  |
| P68032 | ACTC1 |  |  | 5.92E-03 | 1.59 | 1.50E-02 | 1.71 |  |  | 215 |
| Q03591 | CFHR1 |  |  | 2.63E-02 | 0.82 | 2.58E-02 | -0.13 |  |  |  |
| Q06033 | ITIH3 |  |  | 7.40E-03 | 1.15 |  |  |  |  | 58.5 |
| Q14520 | HABP2 |  |  | 8.01E-03 | 0.84 | 2.18E-05 | -0.28 |  |  | 30 |
| Q15848 | ADIPOQ |  |  | 3.41E-03 | 0.71 | 4.56E-04 | -0.29 |  |  |  |
| Q9NZP8 | C1RL |  |  | 2.16E-02 | 0.90 | 4.66E-04 | -0.14 |  |  |  |
| Q9Y5Y7 | LYVE1 |  |  | 4.14E-02 | 0.81 | 2.60E-02 | -0.20 |  |  |  |
| P03952 | KLKB1 |  |  |  |  | 4.56E-04 | -0.12 |  |  | 23.5 |
| P18428 | LBP |  |  |  |  | 1.73E-02 | 0.20 |  | 177 |  |
| P27169 | PON1 |  |  |  |  | 1.04E-02 | -0.07 | 143.5 |  |  |
| P29622 | SERPINA4 |  |  |  |  | 1.34E-02 | -0.11 |  |  | 190 |
| P35858 | IGFALS |  |  |  |  | 3.12E-04 | -0.25 |  |  | 32 |
| P0C0S8 | H2AC11 |  |  |  |  |  |  | 17.5 |  | 21 |
| P48740 | MASP2 |  |  |  |  |  |  | 77.5 | 23.5 |  |
| P61769 | B2M |  |  |  |  |  |  | 95 | 33.5 |  |
| P01593 | IGKV1D-33 |  |  |  |  |  |  | 152 | 167 |  |

^1^ Only proteins represented by minimum two layers of results (significance in univariate statistics or selection as ML feature)

^2^ Ratio of means calculated as Death / Survival.

^3^ Difference calculated as Death – Survival.

^4^ Median rank determined by 100 time Monte-Carlo cross validation.

Supplementary Table 4: Mean performances of the evaluated machine learning models.

Prediction of 30-day survival based on proteome data from day 1

| **Metric (mean)** | **Random Forest** | **Gradient Boost** | **Logistic Regression (Elastic Net)** | **Support Vector Machine** | **Logistic Regression (L1)** |
| --- | --- | --- | --- | --- | --- |
| **Accuracy** | 0.6539 | 0.6004 | 0.6245 | 0.5445 | 0.6171 |
| **Sensitivity** | 0.5792 | 0.6151 | 0.6131 | 0.7800 | 0.6303 |
| **Specificity** | 0.7008 | 0.5911 | 0.6316 | 0.3963 | 0.6089 |
| **Precision** | 0.5525 | 0.4982 | 0.5371 | 0.4570 | 0.5218 |
| **Recall** | 0.5792 | 0.6151 | 0.6131 | 0.7800 | 0.6303 |
| **F1-score** | 0.5624 | 0.5400 | 0.5542 | 0.5687 | 0.5563 |
| **MCC** | 0.2797 | 0.2088 | 0.2531 | 0.1927 | 0.2439 |
| **AUROC** | 0.6963 | 0.6607 | 0.6836 | 0.6897 | 0.6839 |

**Prediction of 4-day survival based on proteome data from day 1**

| **Metric (mean)** | **Random Forest** | **Gradient Boost** | **Logistic Regression (Elastic Net)** | **Support Vector Machine** | **Logistic Regression (L1)** |
| --- | --- | --- | --- | --- | --- |
| **Accuracy** | 0.7343 | 0.7634 | 0.8522 | 0.7054 | 0.5674 |
| **Sensitivity** | 0.5319 | 0.4733 | 0.4040 | 0.5688 | 0.8156 |
| **Specificity** | 0.8423 | 0.8140 | 0.9303 | 0.7783 | 0.4350 |
| **Precision** | 0.6516 | 0.3433 | 0.5688 | 0.6151 | 0.4483 |
| **Recall** | 0.5319 | 0.4733 | 0.4040 | 0.5688 | 0.8156 |
| **F1-score** | 0.5762 | 0.3808 | 0.4481 | 0.5729 | 0.5706 |
| **MCC** | 0.3978 | 0.2609 | 0.3870 | 0.3655 | 0.2565 |
| **AUROC** | 0.7733 | 0.6986 | 0.7260 | 0.7369 | 0.7569 |

**Prediction of 30-day survival using data from days 1 and 4 as well as day 4/ day 1 ratios**

| **Metric (mean)** | **Random Forest** | **Gradient Boost** | **Logistic Regression (Elastic Net)** | **Support Vector Machine** | **Logistic Regression (L1)** |
| --- | --- | --- | --- | --- | --- |
| **Accuracy** | 0.6943 | 0.5914 | 0.4603 | 0.6600 | 0.6312 |
| **Sensitivity** | 0.5924 | 0.6940 | 0.9412 | 0.6684 | 0.6576 |
| **Specificity** | 0.7580 | 0.5273 | 0.1597 | 0.6547 | 0.6148 |
| **Precision** | 0.6115 | 0.5007 | 0.4151 | 0.5621 | 0.5557 |
| **Recall** | 0.5924 | 0.6940 | 0.9412 | 0.6684 | 0.6576 |
| **F1-score** | 0.5957 | 0.5639 | 0.5743 | 0.5975 | 0.5726 |
| **MCC** | 0.3562 | 0.2301 | 0.1414 | 0.3262 | 0.2913 |
| **AUROC** | 0.7461 | 0.6653 | 0.7409 | 0.7395 | 0.7191 |

**Supplementary Table 5:** Feature importance ranks for random forest classifiers.

| **30-day survival based on proteome data from day 1** | | **4-day survival based on proteome data from day 1** | | **30-day survival using proteome data from day 1 and day 4** | |
| --- | --- | --- | --- | --- | --- |
| **Gene** | **Median feature importance rank** | **Gene** | **Median feature importance rank** | **Gene, Data Source** | **Median feature importance rank** |
| LCP1 | 3 | F5 | 4 | MB_day4 | 0 |
| MB | 3 | MST1 | 9 | ALDOA_day4 | 2 |
| F5 | 5 | LCAT | 11 | LDHA_day4 | 7.5 |
| LDHA | 8 | F13B | 11.5 | F13B_day4 | 8 |
| APOF | 9.5 | ACTB | 16.5 | HRG_day4 | 9 |
| CAT | 10 | APOC4 | 18 | MB_day1 | 19 |
| FETUB | 11 | C3 | 18.5 | H2AC17_day4 | 21 |
| TKT | 13 | MASP2 | 23.5 | H2BC12_day4 | 22 |
| H2AC17 | 17.5 | MB | 29.5 | KLKB1_ratios | 23.5 |
| VWF | 20 | VWF | 33 | FETUB_day1 | 24.5 |
| HSPA5 | 21 | B2M | 33.5 | HABP2_ratios | 30 |
| LCAT | 23.5 | PROC | 35.5 | HSPA5_day4 | 30.5 |
| F13B | 24.5 | FETUB | 45.5 | CP_day4 | 31.5 |
| KNG1 | 25 | CST3 | 52 | IGFALS_ratios | 32 |
| CTSD | 25 | CNDP1 | 56.5 | LCP1_day1 | 33 |
| HSP90AA1 | 29 | BTD | 64 | F11_ratios | 33 |
| IGHV169 | 29.5 | CFD | 68.5 | LCAT_day4 | 33.5 |
| HRG | 30.5 | IGHV372 | 75.5 | FETUB_day4 | 41 |
| HBA2 | 37 | APOF | 92.5 | CAT_day4 | 47 |
| ACTB | 40 | A1BG | 97 | PROC_day4 | 51.5 |
| IGKV15 | 40.5 | HBD | 105 | SERPINA6_day4 | 52.5 |
| IGKV116 | 40.5 | IGHM | 106 | VWF_day4 | 56.5 |
| HBB | 44.5 | CLU | 106 | ITIH3_day4 | 58.5 |
| IGKV1D8 | 50 | ALB | 114.5 | TKT_day1 | 63.5 |
| PRDX2 | 50.5 | KNG1 | 116 | F5_day4 | 64.5 |
| ORM1 | 56 | CP | 129 | TKT_day4 | 73 |
| VSIG4 | 64 | HRG | 130.5 | LDHA_day1 | 75 |
| CLEC3B | 68.5 | IGHV439 | 133 | ATRN_day4 | 78 |
| C1QB | 72 | ITIH4 | 138.5 | CLEC3B_day4 | 78.5 |
| F10 | 73.5 | P0DOX2 | 140 | HSP90AA1_day4 | 88 |
| A2M | 74.5 | HBB | 140.5 | F5_day1 | 89 |
| MASP2 | 77.5 | IGHV37 | 148 | LCP1_day4 | 93 |
| PLG | 80 | KRT1 | 155.5 | KNG1_day1 | 95.5 |
| CD5L | 84 | GPX3 | 155.5 | IGLV469_ratios | 106.5 |
| C3 | 88 | IGLV861 | 156.5 | LYZ_ratios | 109.5 |
| B2M | 95 | LGALS3BP | 158.5 | ALDOB_day1 | 124 |
| VTN | 101.5 | IGHV374 | 161 | IGKV3D20_day4 | 128.5 |
| IGKV18 | 101.5 | IGKV1D-33 | 167 | SERPINA4_ratios | 190 |
| PON1 | 143.5 | LBP | 177 | HPR_day4 | 190 |
| IGKV1D-33 | 152 | CA2 | 192.5 | ACTC1_day4 | 215 |

Each classifier was trained 100 times in a Monte Carlo cross-validation and the features were interpreted using Shapley Additive Explanations (SHAP).

**Supplementary Table 6:** Numbers of survivors and non-survivors for the study of Mi et al.

| **Time point in ICU** | **non-survivors (n)** | **Survivors (n)** | **p_FDR_ ≤ 0.05** |
| --- | --- | --- | --- |
| Day1 | 96 | 493 | 43 |
| Day3 | 123 | 577 | 31 |
| Day5 | 99 | 459 | 35 |

**Supplementary Table 7:** Significantly differentially abundant proteins from the study of Mi et al.

**Day 1**

| **Protein** | **Gene** | **Adjusted p-value** | **Ratio of means**  **(Death /Survival)** |
| --- | --- | --- | --- |
| P04004 | VTN | 2.56E-04 | 0.80 |
| P00747 | PLG | 1.77E-03 | 0.85 |
| P05546 | SERPIND1 | 1.77E-03 | 0.89 |
| P00738 | HP | 1.77E-03 | 0.75 |
| P07360 | C8G | 1.77E-03 | 0.90 |
| P55056 | APOC4 | 1.77E-03 | 0.79 |
| Q08380 | LGALS3BP | 1.77E-03 | 1.45 |
| P04196 | HRG | 1.83E-03 | 0.88 |
| P05109 | S100A8 | 2.46E-03 | 1.50 |
| P01008 | SERPINC1 | 2.46E-03 | 0.90 |
| P27169 | PON1 | 3.25E-03 | 0.85 |
| P19320 | VCAM1 | 3.25E-03 | 1.26 |
| P01042 | KNG1 | 3.82E-03 | 0.90 |
| P02774 | GC | 3.82E-03 | 0.88 |
| P04070 | PROC | 3.82E-03 | 0.84 |
| P05156 | CFI | 7.94E-03 | 0.89 |
| P0DOY2 | IGLC2 | 1.15E-02 | 1.18 |
| P02671 | FGA | 1.15E-02 | 0.88 |
| P02786 | TFRC | 1.31E-02 | 1.12 |
| A0A0C4DH31 | IGHV1-18 | 1.55E-02 | 1.13 |
| P02647 | APOA1 | 1.72E-02 | 0.86 |
| P12109 | COL6A1 | 2.01E-02 | 1.44 |
| P80188 | LCN2 | 2.01E-02 | 1.42 |
| P59665 | DEFA1 | 2.01E-02 | 1.23 |
| Q9UGM5 | FETUB | 2.15E-02 | 0.84 |
| P01880 | IGHD | 3.03E-02 | 1.35 |
| P19823 | ITIH2 | 3.07E-02 | 0.91 |
| P01782 | IGHV3-9 | 3.07E-02 | 1.24 |
| P06702 | S100A9 | 3.18E-02 | 1.23 |
| P24821 | TNC | 3.19E-02 | 1.42 |
| P05452 | CLEC3B | 3.19E-02 | 0.89 |
| Q9BYV9 | BACH2 | 4.27E-02 | 0.71 |
| P15169 | CPN1 | 4.27E-02 | 0.92 |
| Q8N1H7 | SIX6OS1 | 4.48E-02 | 1.22 |
| P01602 | IGKV1-5 | 4.48E-02 | 1.19 |
| P16070 | CD44 | 4.76E-02 | 1.21 |
| P80748 | IGLV3-21 | 4.76E-02 | 1.36 |
| P02765 | AHSG | 4.76E-02 | 0.90 |
| P05154 | SERPINA5 | 4.88E-02 | 0.83 |
| P19827 | ITIH1 | 4.88E-02 | 0.92 |
| Q15582 | TGFBI | 4.88E-02 | 1.14 |
| P06681 | C2 | 4.93E-02 | 0.93 |
| P08697 | SERPINF2 | 4.93E-02 | 0.92 |

**Day 3**

| **Protein** | **Gene** | **Adjusted p-value** | **Ratio of means**  **(Death /Survival)** |
| --- | --- | --- | --- |
| P00738 | HP | 1.46E-05 | 0.73 |
| P02790 | HPX | 1.32E-04 | 0.86 |
| P55056 | APOC4 | 1.32E-04 | 0.78 |
| P04004 | VTN | 1.32E-04 | 0.83 |
| P01008 | SERPINC1 | 3.68E-04 | 0.89 |
| P01782 | IGHV3-9 | 3.78E-04 | 1.29 |
| P13765 | HLA-DOB | 3.78E-04 | 0.83 |
| P04196 | HRG | 8.23E-04 | 0.90 |
| P05546 | SERPIND1 | 2.74E-03 | 0.91 |
| P03952 | KLKB1 | 3.91E-03 | 0.90 |
| P01042 | KNG1 | 9.63E-03 | 0.91 |
| P02766 | TTR | 1.01E-02 | 0.87 |
| P06702 | S100A9 | 1.01E-02 | 1.25 |
| P07360 | C8G | 1.23E-02 | 0.93 |
| Q6UX71 | PLXDC2 | 1.44E-02 | 1.17 |
| P04275 | VWF | 1.68E-02 | 1.25 |
| P05109 | S100A8 | 1.68E-02 | 1.38 |
| O75460 | ERN1 | 1.83E-02 | 0.91 |
| P00734 | F2 | 1.83E-02 | 0.93 |
| P02748 | C9 | 1.83E-02 | 0.91 |
| P61769 | B2M | 1.83E-02 | 1.23 |
| P80511 | S100A12 | 2.20E-02 | 1.59 |
| P02774 | GC | 2.20E-02 | 0.91 |
| P02652 | APOA2 | 2.28E-02 | 0.85 |
| P13671 | C6 | 2.40E-02 | 0.90 |
| P06727 | APOA4 | 2.58E-02 | 1.14 |
| Q6ZSZ5 | ARHGEF18 | 2.59E-02 | 0.76 |
| P19320 | VCAM1 | 2.78E-02 | 1.19 |
| P00747 | PLG | 2.78E-02 | 0.92 |
| P05452 | CLEC3B | 2.84E-02 | 0.91 |
| P02743 | APCS | 2.85E-02 | 0.92 |

**Day 5**

| **Protein** | **Gene** | **Adjusted p-value** | **Ratio of means**  **(Death /Survival)** |
| --- | --- | --- | --- |
| P05546 | SERPIND1 | 8.45E-06 | 0.87 |
| P04196 | HRG | 4.41E-05 | 0.86 |
| P03952 | KLKB1 | 4.41E-05 | 0.87 |
| P43652 | AFM | 3.03E-04 | 0.85 |
| P00747 | PLG | 3.08E-04 | 0.86 |
| P02790 | HPX | 3.09E-04 | 0.86 |
| P04114 | APOB | 5.01E-04 | 0.83 |
| P19823 | ITIH2 | 9.17E-04 | 0.88 |
| P15291 | B4GALT1 | 1.95E-03 | 1.51 |
| P01031 | C5 | 2.06E-03 | 0.91 |
| P05109 | S100A8 | 2.68E-03 | 1.52 |
| O95445 | APOM | 2.95E-03 | 0.70 |
| P01008 | SERPINC1 | 2.95E-03 | 0.89 |
| P24821 | TNC | 4.47E-03 | 1.55 |
| P06702 | S100A9 | 4.47E-03 | 1.27 |
| P25311 | AZGP1 | 6.02E-03 | 1.23 |
| P41222 | PTGDS | 7.95E-03 | 1.22 |
| P01034 | CST3 | 9.03E-03 | 1.21 |
| P06727 | APOA4 | 9.03E-03 | 1.20 |
| P51884 | LUM | 9.03E-03 | 1.10 |
| P02656 | APOC3 | 9.03E-03 | 0.81 |
| P02766 | TTR | 9.38E-03 | 0.85 |
| P55056 | APOC4 | 1.02E-02 | 0.83 |
| P29622 | SERPINA4 | 1.02E-02 | 0.85 |
| P01024 | C3 | 1.21E-02 | 0.89 |
| P01042 | KNG1 | 1.29E-02 | 0.90 |
| P0C0L5 | C4B | 1.46E-02 | 0.84 |
| P02655 | APOC2 | 1.72E-02 | 0.87 |
| P08185 | SERPINA6 | 2.06E-02 | 0.92 |
| P0DJI9 | SAA2 | 2.07E-02 | 1.32 |
| P00746 | CFD | 2.39E-02 | 1.17 |
| P12259 | F5 | 2.86E-02 | 0.82 |
| P00751 | CFB | 4.13E-02 | 0.90 |
| P01721 | IGLV6-57 | 4.17E-02 | 1.13 |
| Q96PD5 | PGLYRP2 | 4.62E-02 | 0.89 |

**Supplementary Figures**

**
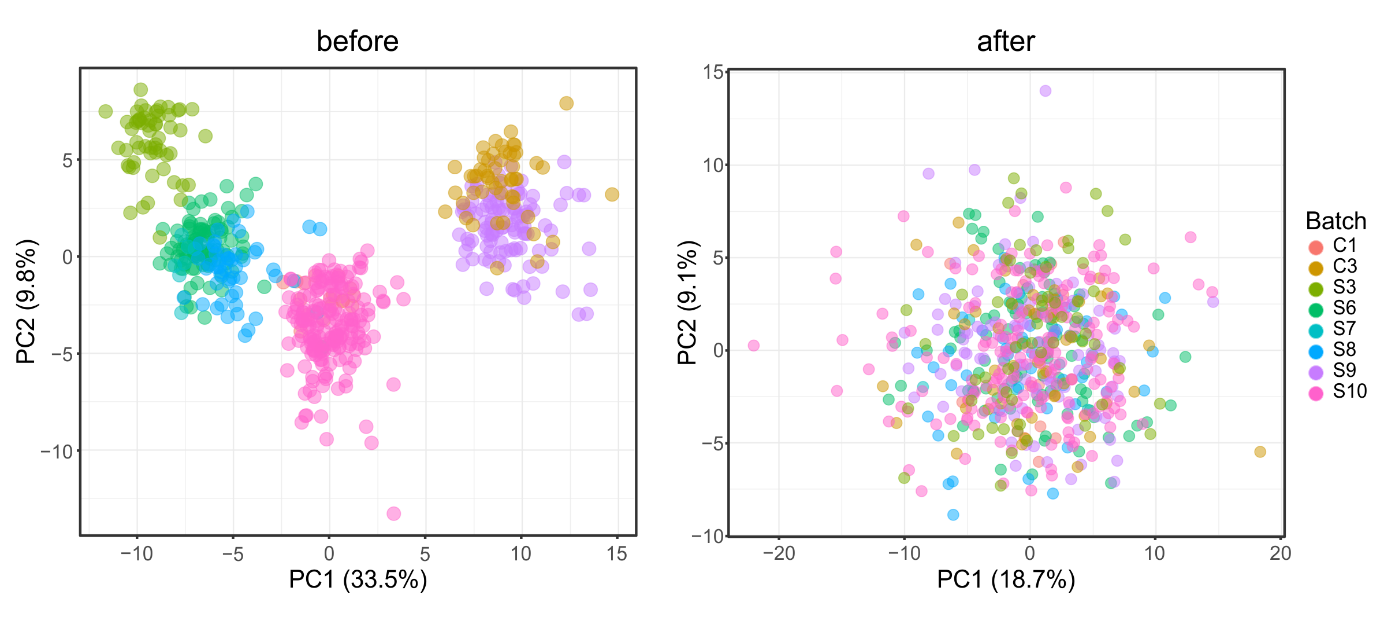
Supplementary Figure 1:** Principal component analysis (PCA) plots of mass spectrometry data before and after batch normalization. Each data point corresponds to a sample, colors representing the respective batches.

**
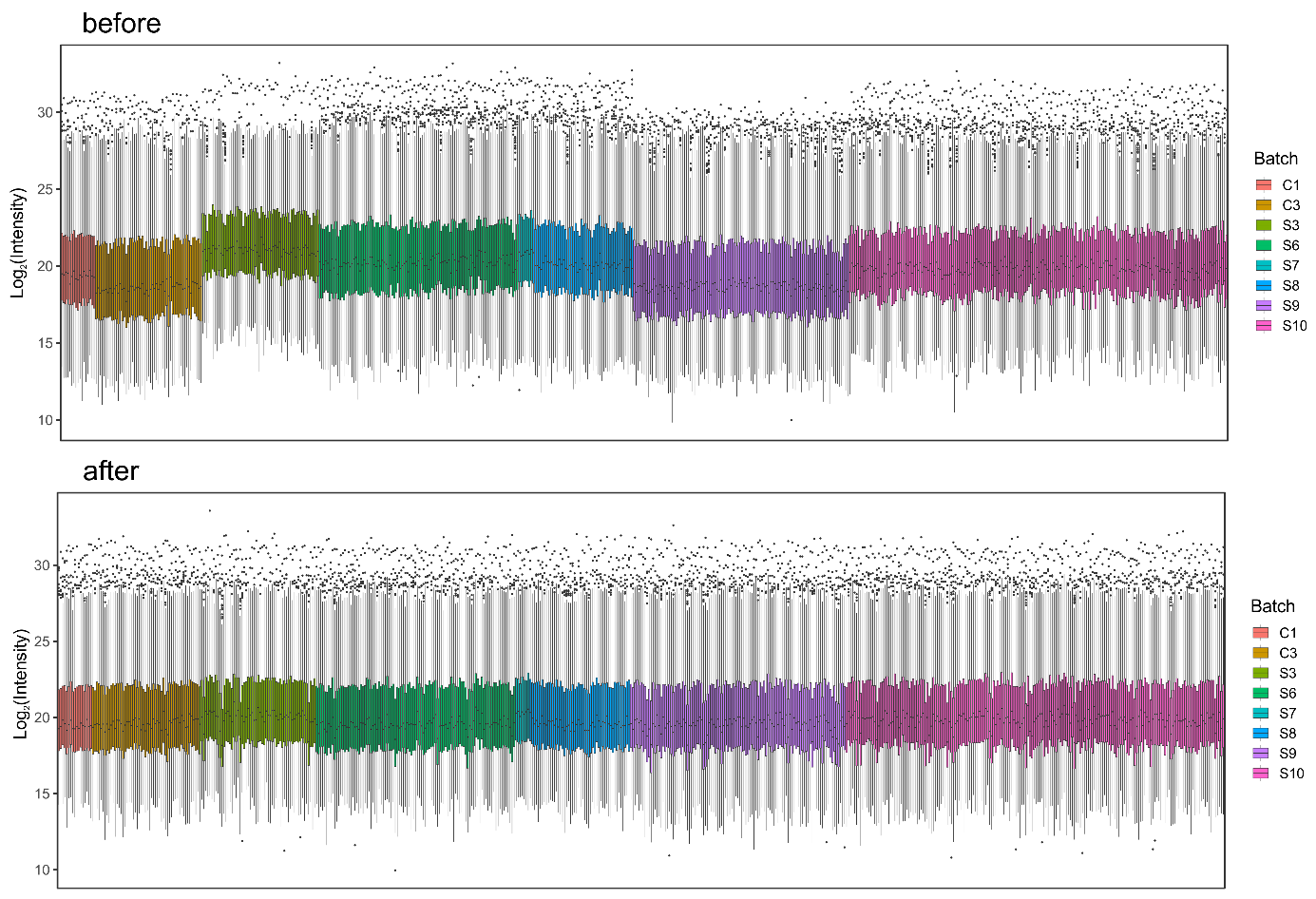
**

**Supplementary Figure 2:** Boxplot representation of protein intensities before and after normalization. Boxes indicate the 25% - 75% interquartile range (IQR) with the median displayed as horizontal line. Whiskers extend to 1.5 x IQR. Outliers displayed as individual data points. Each box corresponds to a sample, colors representing the respective batches.


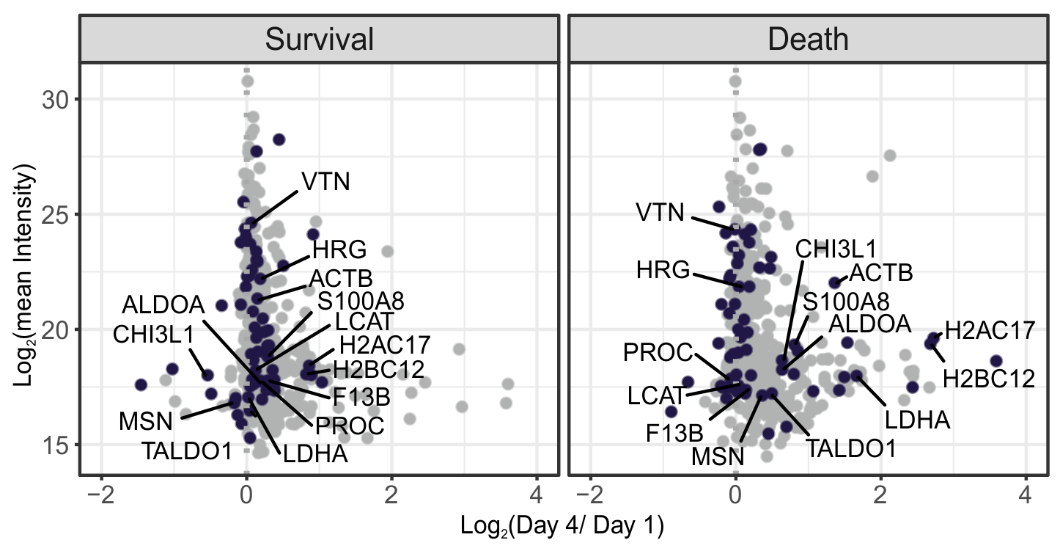


**Supplementary Figure 3:** Scatter plots (MA plots) showing the relative changes in protein intensities between day 1 and day 4 (calculated day 4/ day 1) separately for deceased and survived patients. Significant proteins colored (p_FDR_ value ≤ 0.05, t-test, Benjamini-Hochberg corrected). The 14 proteins that were found to be significant in all three univariate analyses are labeled with gene names.


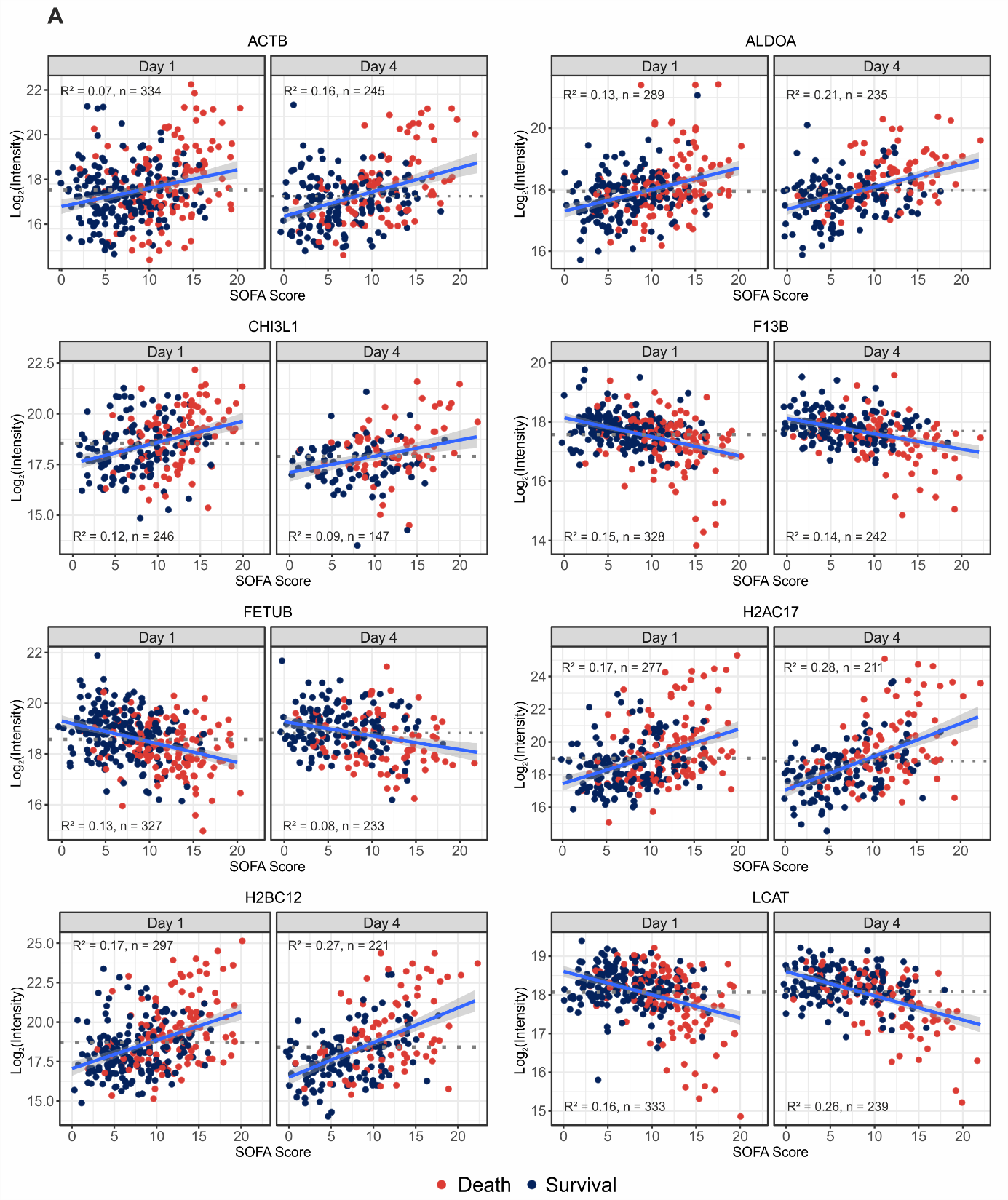

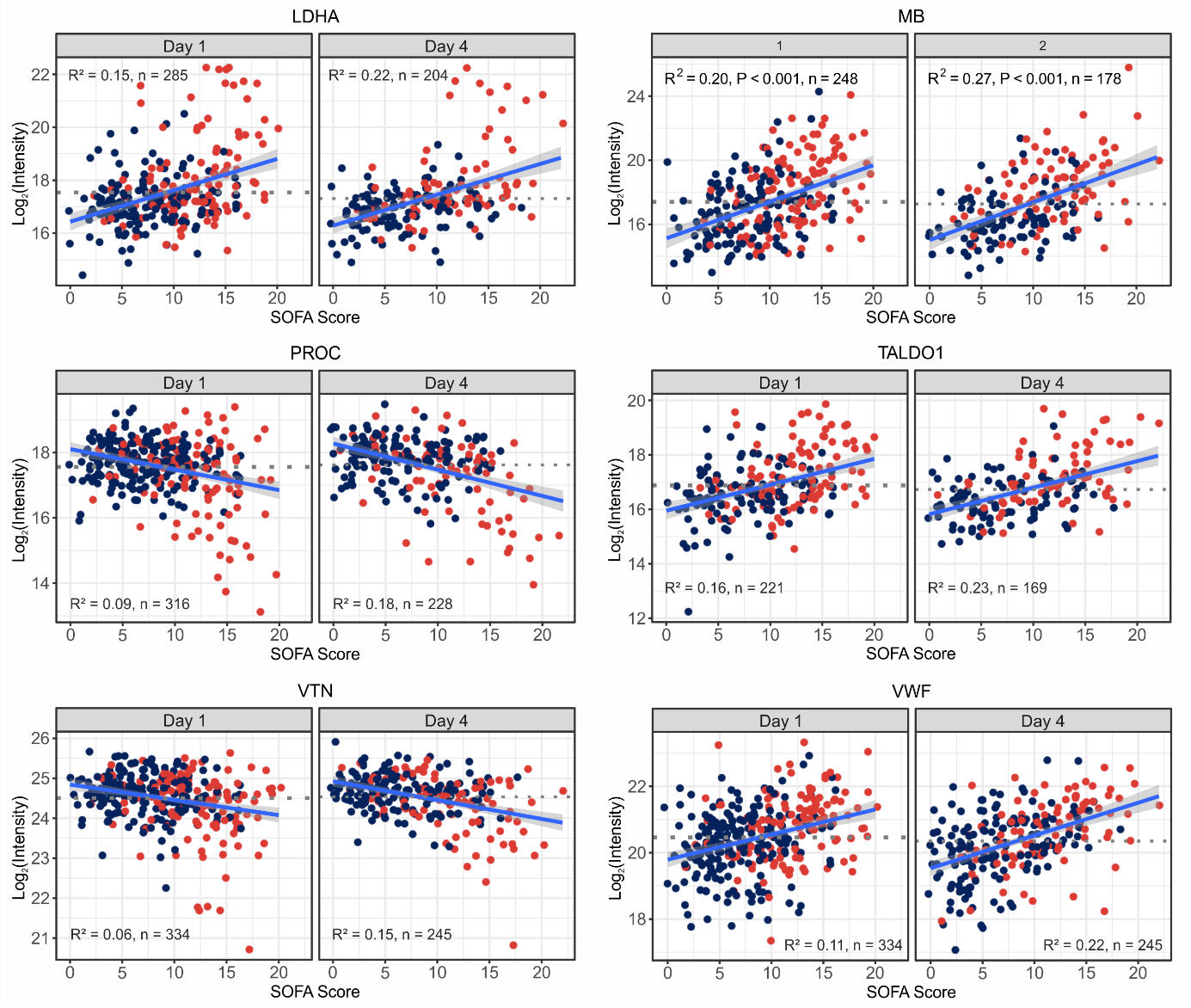

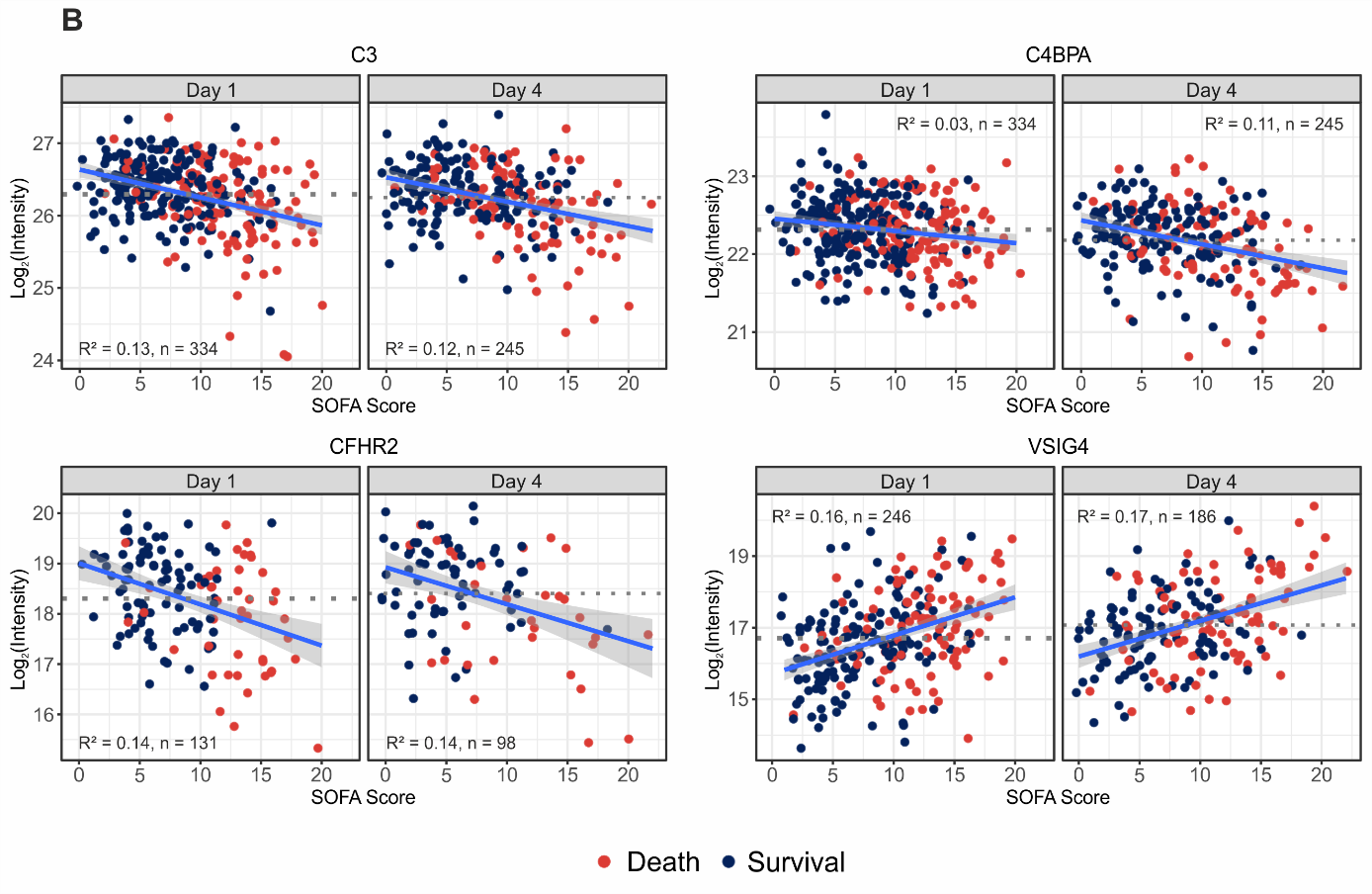


**Supplementary Figure 4:** Linear regression analysis was performed for all significantly regulated proteins with the SOFA score separately for days 1 and 4. Proteins were filtered for a minimum R² of 0.1 for at least one time point. **(A)** The examples shown here, were overlapping between all three univariate tests or selected by all three ML classifiers. **(B)** Additional proteins that were associated with complement activation. Blue and red data points representing survived and deceased patient, respectively. Blue lines representing the linear fit with its confidence interval. All displayed correlations were significant with p < 0.001.
